# Generating Dynamic Structures Through Physics‐Based Sampling of Predicted Inter‐Residue Geometries

**DOI:** 10.1002/advs.202518469

**Published:** 2026-01-21

**Authors:** Chenxiao Xiang, Wenkai Wang, Zhenling Peng, Jianyi Yang

**Affiliations:** ^1^ MOE Frontiers Science Center for Nonlinear Expectations, State Key Laboratory of Cryptography and Digital Economy Security, Research Center for Mathematics and Interdisciplinary Sciences Shandong University Qingdao China

**Keywords:** deep learning, protein structure prediction, protein dynamic structures

## Abstract

Deep learning‐based methods, such as AlphaFold2, have revolutionized the prediction of static protein structures. However, modeling alternative conformations and dynamic structures remains an unsolved problem. Here, we present trRosettaX2‐Dynamics (trX2‐D), an innovative solution building on our CASP15 and CASP16 winning method, trRosettaX2. trX2‐D tackles this challenge by employing physics‐based iterative sampling of trRosettaX2's predicted inter‐residue geometric distributions. The model underwent pre‐training on high‐resolution X‐ray structures, followed by fine‐tuning on approximately 7000 dynamic NMR structures. This dual training regime significantly bolsters its capacity to predict alternative conformations and dynamic structures. At its core, trX2‐D employs a Transformer‐based neural network to initially predict a set of inter‐residue geometric constraints. These constraints are then iteratively sampled to generate dynamic structures, entirely circumventing the need for prior knowledge of native structural states. Extensive benchmarking across three distinct datasets—two focused on alternative conformations and one on dynamic structures—demonstrates trX2‐D's promising ability to predict alternative conformations and accurately capture structural dynamics. This work highlights the potential of integrating deep learning predictions with physics‐based sampling to advance the field of protein dynamic structure prediction.

## Graphical abstract

While static structure prediction has been revolutionized, modeling protein dynamics remains elusive. trRosettaX2‐Dynamics is presented to address this challenge. This framework leverages a Transformer‐based network to predict inter‐residue geometric constraints, guiding conformation generation via physics‐based iterative sampling. The resulting method effectively explores alternative conformations without requiring prior knowledge of native states.

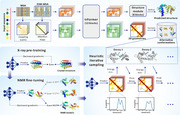

## 1 Introduction

Protein structures are fundamental determinants of biological function [1], and their dynamic conformational changes orchestrate many cellular processes [2]. Understanding their conformational landscape is crucial for deciphering mechanisms of biological action, developing therapeutics, and engineering novel biological molecules. While deep learning approaches, such as AlphaFold2 (AF2) [3], RoseTTAFold [4], trRosetta [5, 6, 7, 8], and ESMFold [9], have revolutionized static protein structure prediction, accurately modeling alternative conformations of proteins remains a significant challenge.

The challenge in predicting multiple conformations arises from the scarcity of experimental data. Recently, Bryant et al. [10] revealed a surprisingly small number of sequence clusters (<1000) exhibiting significant conformational diversity in the Protein Data Bank (PDB) [11]. Consequently, current data‐driven methods like AF2 tend to produce conformations that closely resemble those experimentally resolved in the PDB, posing a challenge to effectively capturing the protein conformational diversity.

Several strategies have been explored to address the challenge of multi‐conformation prediction [12]. Methods leveraging contact maps of a known conformation to predict another have demonstrated the ability to generate alternative conformations [13]. However, these methods are inherently limited as they require prior knowledge of at least one native conformational state and struggle to predict more than two distinct conformations. Molecular dynamics simulations offer a physics‐based approach to explore the conformational landscape of proteins [14], but the substantial computational cost and time requirements limit their applicability to large protein systems. The AF2‐based approaches, such as AF‐Cluster [15], have shown promise in generating multiple conformations by clustering and sampling diverse inputs for AF2 (e.g., multiple sequence alignment (MSA) and template [10, 15, 16]). Nevertheless, concerns regarding the true performance of these AF2‐based methods have emerged due to inadequate benchmark testing and data leakage stemming from the AF2 training set [10, 17]. To address this issue, Cfold [10] retrained and evaluated AF2 using a meticulously constructed training‐test split, and employed MSA clustering and random dropout to generate diverse conformations. Nevertheless, their results indicated that certain conformations remain elusive using MSA clustering and random dropout strategies.

Overall, AF‐Cluster and other deep learning methods rely on modifying the inputs to the model in an attempt to generate multiple conformations. However, the effectiveness of these input perturbation strategies depends on highly informative inputs. For example, AF‐Cluster struggles with shallow MSAs (e.g., depth < 10). Moreover, lacking direct control over the predicted structures might limit the diversity and functional relevance of the generated conformations.

Building on this observation, we introduce trRosettaX2‐Dynamics (trX2‐D), a novel deep learning‐based approach to predict multiple conformations using an output‐driven iterative sampling strategy. This method is primarily powered by a Transformer‐based protein structure prediction method, trRosettaX2 (trX2), which is an improved version of trRosettaX [7] and outperforms RoseTTAFold, though using much fewer parameters and computational resources [18]. trX2 adopts an end‐to‐end architecture that can simultaneously predict the 2D geometries (1 distance and 3 orientations defined in trRosetta [5]) and 3D structures. A unique property of the predicted 2D geometries lies in that they are represented as probability distributions and thus potentially encode latent information about alternative conformations. Inspired by this, trX2‐D designs a heuristic module to sample diverse conformations based on the iterative sampling of the predicted 2D geometries, which allows the generation of multiple conformations without any prior information. In addition, trX2‐D employs a fine‐tuning strategy on the dynamic structures solved by Nuclear Magnetic Resonance (NMR) experiments to improve the conformational diversity information in the predicted geometries.

We evaluated trX2‐D on three datasets non‐redundant to its training set, including two established benchmarks for dual‐conformation proteins and a dataset of dynamic proteins. Benchmark tests show that trX2‐D significantly improves upon the performance of the base trX2 model and shows promise in predicting alternative conformations on dual‐conformation benchmarks. Furthermore, our tests on the dynamic protein dataset indicate that trX2‐D can generate more diverse conformation ensembles compared to other methods. In summary, trX2‐D represents a novel and promising approach for predicting protein alternative conformations, marking a solid step towards a more comprehensive understanding of protein structural dynamics.

## 2 Results

### 2.1 Overview of the Method

**FIGURE 1:**
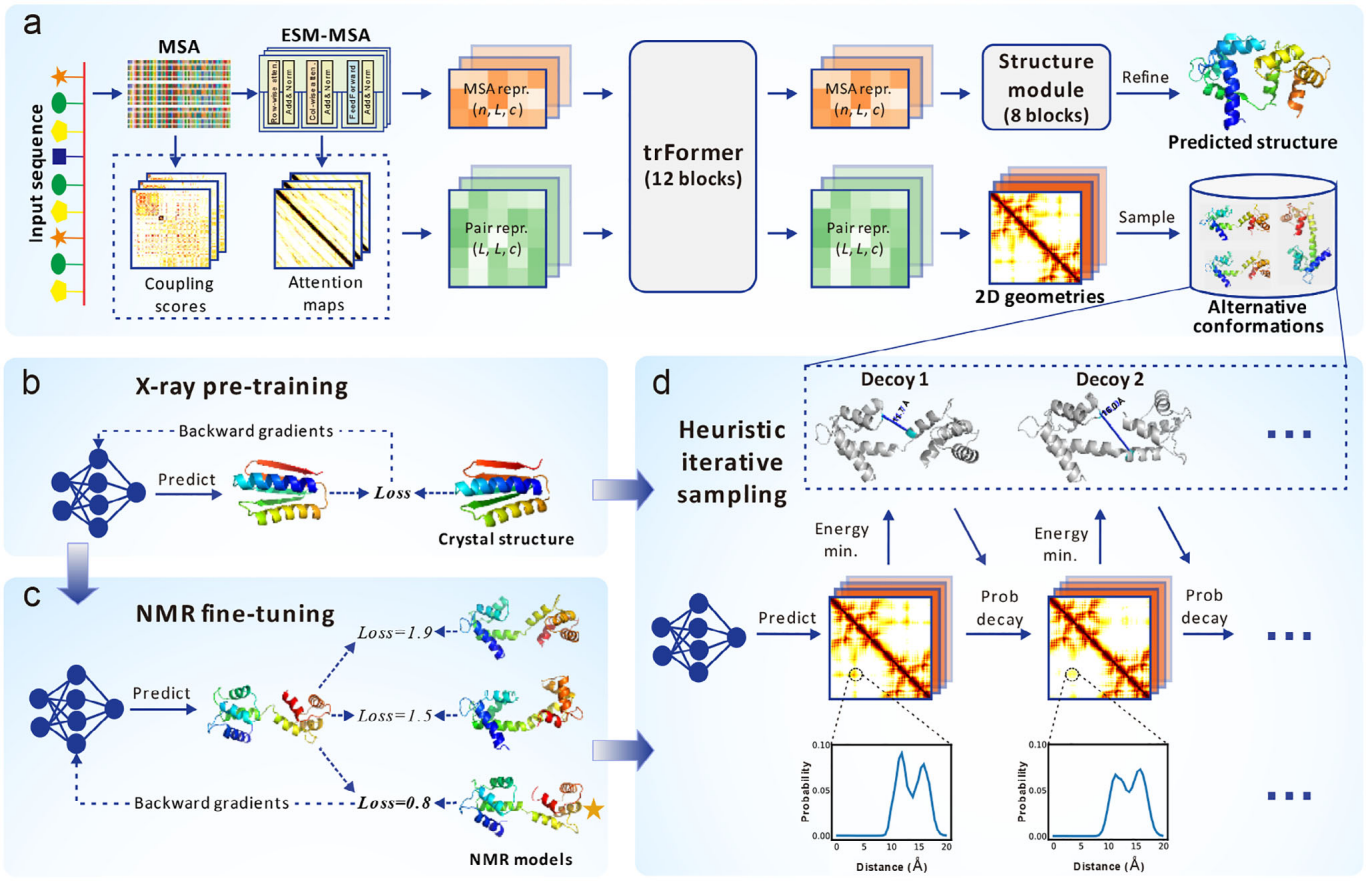
Architectures of trRosettaX2 and trRosettaX2‐Dynamics. (a) overview of trRosettaX2 and trRosettaX2‐Dynamics. The sole input is the amino acid sequence of a target protein. A multiple sequence alignment (MSA) is generated and converted into two representations, MSA representation and pair representation, which are updated through a Transformer‐based module (trFormer). The updated representations are fed into the structure module to predict the static 3D structure by trRosettaX2. Meanwhile, 2D geometries derived from the pair representation are used to sample alternative conformations by trRosettaX2‐Dynamics. *n*, *L*, and *c* refer to the number of MSA rows, sequence length, and number of channels (128 here), respectively. (b,c) trRosettaX2‐Dynamics is first pre‐trained with X‐ray structures (b) and then fine‐tuned with NMR structures (c). (d) The iterative sampling of alternative conformations using predicted 2D geometries.

trRosettaX2 (trX2) is a lightweight protein structure prediction algorithm designed to achieve competitive performance using limited computational resources, which has been briefly introduced before [18]. As shown in Figure 1a, trRosettaX2 employs a Transformer‐based neural network, trFormer, to predict 2D geometries (distance and orientations) from multiple sequence alignment (MSA). The 3D structure is then folded through either structure module (i.e., end‐to‐end prediction) or energy minimization (i.e., two‐step prediction). Although the accuracy of trX2 still slightly lags behind that of AlphaFold2 (AF2), its unique advantages, such as rapid MSA selection and the generation of decoys complementary to the AF2 predictions, helped our group win the championship in CASP15 [18, 19] and CASP16 experiments (https://predictioncenter.org/casp16/zscores_final.cgi). For the detailed methodology description and performance analysis of trX2, please refer to Text S1.

Building upon trRosettaX2, we developed trRosettaX2‐Dynamics (trX2‐D) to improve protein conformation generation. This advancement incorporates two principal modifications: 1) fine‐tuning trX2 with NMR ensembles (trX2 (NMR); see Figure 1b,c and Methods for details) to enhance the representation of dynamic signals within the model outputs; and 2) designing an iterative process for sampling diverse 2D geometries to generate multiple distinct conformations (see Figure 1d; Figure S2, and Methods for details). The complete trX2‐D workflow leverages both the original trX2 and the NMR fine‐tuned trX2 (NMR) in parallel to produce two sets of initial 2D geometry predictions. These predictions subsequently serve as inputs to the iterative process, yielding a diverse ensemble of protein conformations.

### 2.2 Performance of trX2‐D in Distinguishing Apo and Holo States

We evaluated the performance of trX2‐D using an elaborately collected dataset of 91 proteins, which were experimentally solved in apo‐holo states [13, 20]. To focus our analysis on substantial conformational changes, subsequent detailed analyses centered on a subset of 37 proteins exhibiting large conformational changes (see Methods for details) [10, 13]. The information on these conformation pairs is listed in Table S2. The results for the remaining samples are detailed in Supporting Information, which lead to similar conclusions.

**FIGURE 2:**
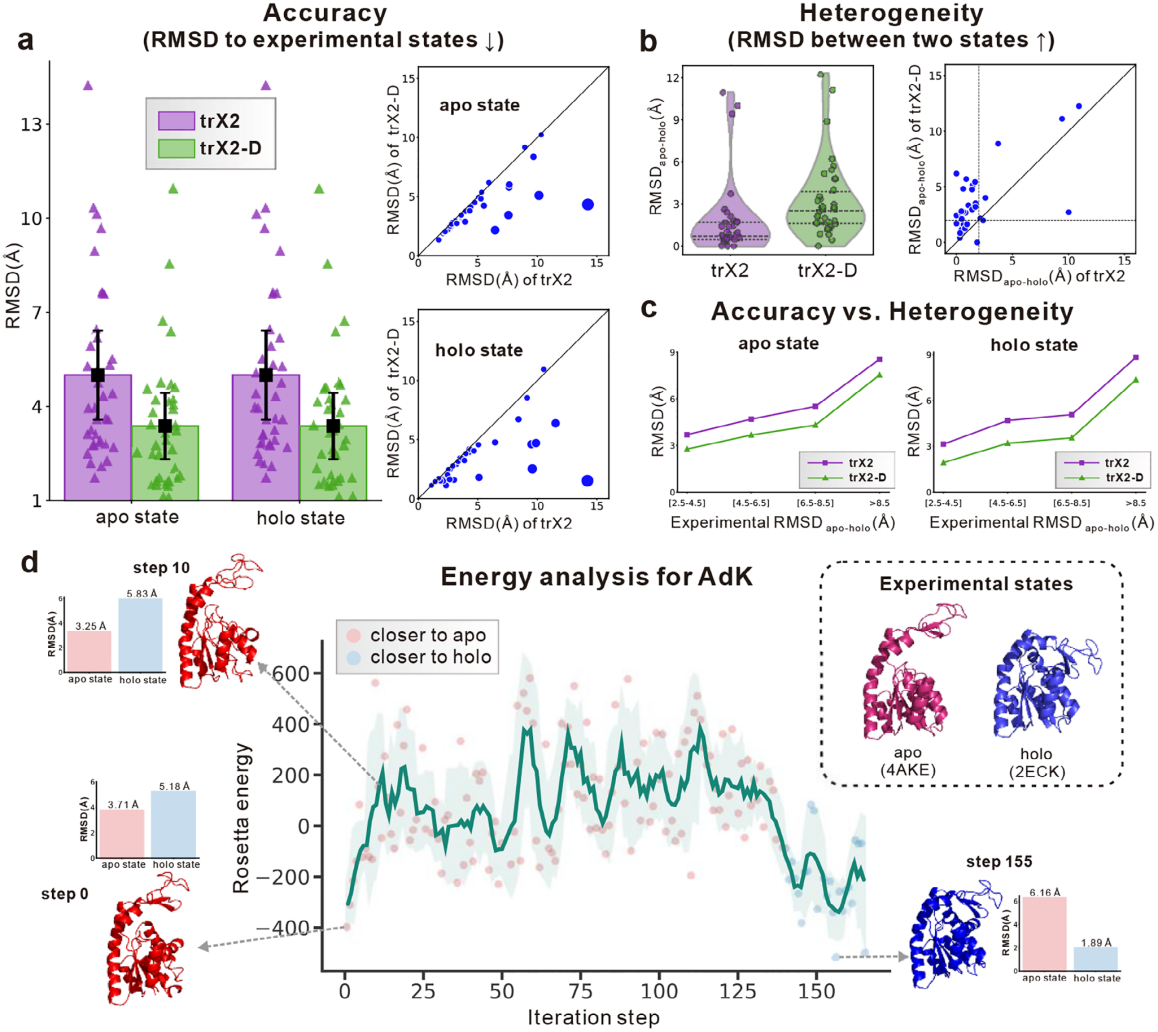
Performance of trX2‐D on the apo‐holo benchmarks. (a) Accuracy assessment measured by RMSD relative to experimental reference structures. Bars represent mean values, and error bars denote half of the standard deviations. Individual points overlaying the bars represent specific targets. In the head‐to‐head scatter plots, point sizes are proportional to the absolute performance difference between the two methods. (b) Evaluation of conformational heterogeneity, quantified by the pairwise RMSD between the two predicted states (RMSD_apo‐holo_). In the violin plots, the internal lines represent the quartiles (dashed) and median (solid). Dashed vertical and horizontal lines in the scatter plot mark a structural similarity cutoff of 2 Å. Arrows in the titles of (a) and (b) denote the direction of better performance. (c) Correlation between prediction accuracy and the magnitude of experimental conformational change (RMSD_apo‐holo_). (d) Energy landscape analysis of a representative target, Adenylate Kinase (AdK). Predicted conformations are colored by state assignment (red: apo; blue: holo); corresponding RMSD values are indicated next to each structure.

We first compare trX2‐D with the default trX2 model to examine the extent of improvement in capturing alternative conformations. In this work, we use RMSD as the primary evaluation metric, which better reflects local structural variation than the TM‐score. A supplementary TM‐score comparison, consistent with the RMSD findings, is provided in Figure S3. Figure 2a illustrates the overall performance of trX2‐D and trX2 predictions for both apo and holo states. The default trX2 produces higher average RMSDs (∼4.8 Å) for both apo and holo state predictions. As shown in Figure S4, trX2 predictions exhibit similar RMSD values relative to both the apo and holo states, suggesting they may represent intermediate states that deviate from the apo and holo conformations. In contrast, by sampling diverse 2D geometries, trX2‐D demonstrates the capability to transition from these trX2‐predicted intermediate states towards either the apo or holo state, consequently yielding enhanced predictive stability. As a result, trX2‐D achieved significantly lower RMSD values (a 20%∼30% reduction) compared to trX2 for both states, highlighting its effectiveness in improving the alternative conformation generation.

The benefits of trX2‐D are further underscored by a direct head‐to‐head RMSD comparison with trX2 for both apo and holo states (Figure 2b). A clear majority of data points (34/37 for the apo state; 33/37 for the holo state) fall below the diagonal line, which means that trX2‐D achieves lower RMSD for ∼ 90% of samples in both states. This trend is particularly pronounced for samples where the original trX2 performed poorly (RMSD > 5 Å).

Beyond improving accuracy, trX2‐D significantly enhances conformational heterogeneity, which is measured by the RMSD between predicted apo and holo states (denoted as RMSD_apo‐holo_). As shown in Figure 2b, trX2 models are largely homogeneous (average ∼1.7 Å), with 30/37 targets showing < 2 Å deviation. Conversely, trX2‐D captures greater heterogeneity (average ∼3.2 Å), limiting high‐similarity cases (< 2 Å) to only 15/37 (40%), a marked improvement over trX2.

To gain further insight into the factors underlying this enhanced performance, we analyzed the influence of experimental conformational heterogeneity on the prediction accuracy of the trX2 model. Our analysis revealed that the RMSD of trX2 predictions positively correlates with the divergence between experimental states (i.e., RMSD_apo‐holo_), with a Pearson correlation coefficient (PCC) of 0.58 (blue line in Figure S5). This finding indicates that heterogeneity between experimental states tends to pose challenges to trX2's accurate prediction. This trend is more pronounced for 16 samples exhibiting conformational differences of RMSD_apo‐holo_ > 5 Å between their experimental states, where the PCC increases to 0.84 (orange line in Figure S5). This highlights trX2's difficulty in accurately modeling cases with significant structural variability. Despite this challenge faced by trX2, trX2‐D achieves consistent improvements across all levels of conformational divergence (Figure 2c). This result further confirms the robust improvement made by trX2‐D.

### 2.3 Interpretability via the Energy Landscape

As an output‐driven method that incorporates physical energy, trX2‐D offers a unique advantage in the interpretability of the energy landscape. Unlike “black‐box” input‐driven approaches, trX2‐D generates diverse conformational ensembles that provide mechanistic insight into transition pathways. We illustrate this capability using Adenylate Kinase (AdK), a classic system characterized by a large‐scale transition between an apo state (PDB ID: 4AKE) and a holo state (PDB ID: 2ECK).

The observed trajectory reveals a compelling physical mechanism for conformational switching. As shown in Figure 2d, the initial predicted structure starts closer to the apo state (RMSD: 3.71 Å) than the holo state (5.18 Å). The early phase of the iteration exhibits a sharp spike in Rosetta energy, which aligns with recent findings on energetic frustration [21]: the transition between conformational states requires an initial destabilization of the starting state by disrupting specific intramolecular interactions. This process results in a high‐energy “activated” conformation (step 10) where key stabilizing factors (e.g., hydrogen bonds or salt bridges holding the apo geometry) are disrupted. Consequently, this conformation exhibits increased local flexibility and loop formation, consistent with the “cracking” mechanism where regions become transiently disordered to facilitate barrier crossing [21]. Interestingly, this destabilization allows the structure to adopt a slightly more open topology, yielding a decreased RMSD relative to the apo state (RMSD: 3.25 Å) before the transition proceeds.

Subsequently, the energy fluctuates as the system explores the conformational space, attempting to escape the local energy minimum of the apo basin. Around step 130, the system successfully surmounts the energy barrier, marked by a distinct drop in energy, and transitions toward the holo state (with step 140 marking the boundary). Finally, at step 155, the energy converges to its lowest value, which remarkably coincides with a conformation highly resembling the native holo structure (RMSD: 1.89 Å).

The interpretability based on the energy landscape, combined with our iterative sampling strategy, offers valuable insights into the dynamics of conformational switching. This suggests a promising avenue for approaching more complex challenges, including the characterization of transient intermediate states.

### 2.4 Impact of NMR‐Based Fine‐Tuning and Iterative Sampling

**FIGURE 3:**
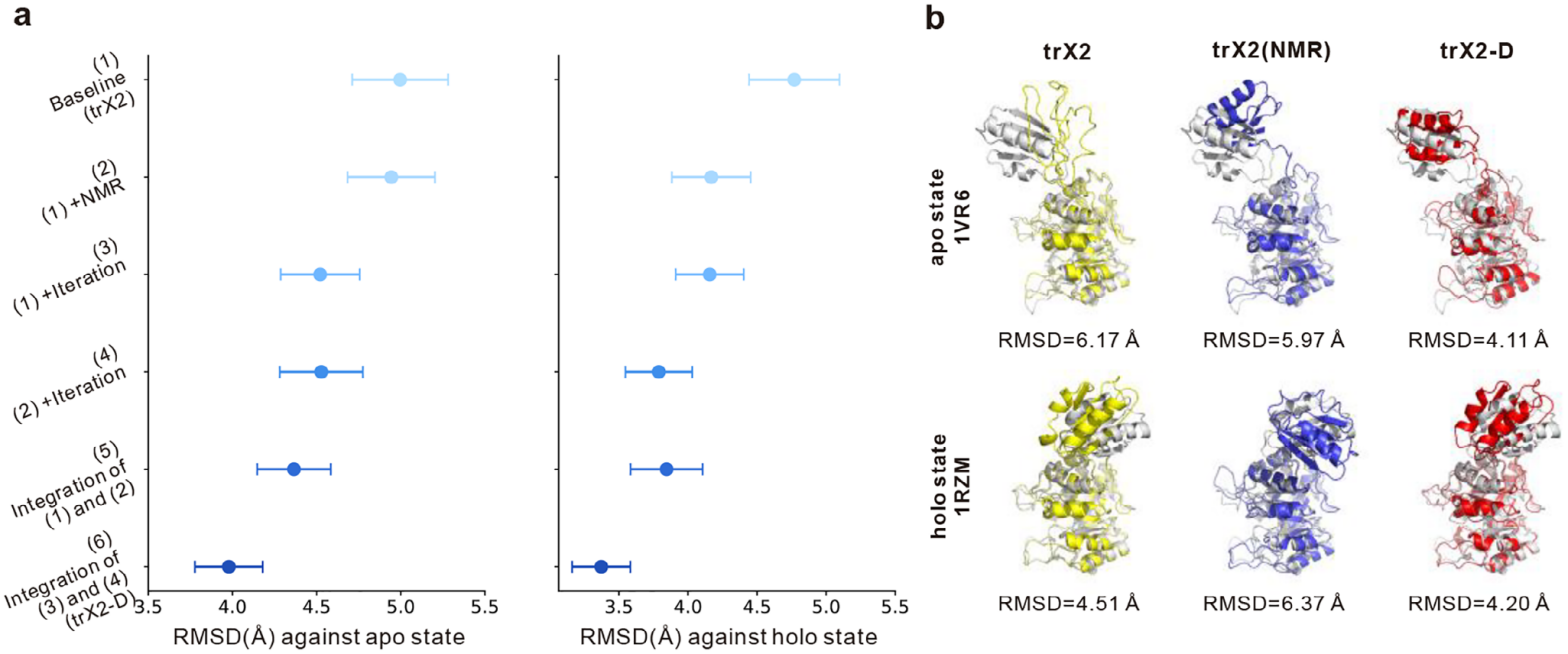
Ablation study on the apo‐holo benchmarks. (a) Average RMSD of predicted conformations across both states. Error bars denote one‐tenth of the standard deviation across targets. (b) Predicted structures for a representative target exhibiting significant inter‐domain rotation (apo: 1VR6; holo: 1RZM). Relative to the trX2 baseline, trX2 (NMR) improves Apo prediction but compromises Holo accuracy. In contrast, trX2‐D enhances predictions for both states, effectively recapitulating the domain rotation.

trX2‐D leverages the architecture of the trX2 network. To enhance its ability to generate diverse conformations, we employed fine‐tuning on a dataset of dynamic structures derived from NMR experiments [22]. Moreover, trX2‐D introduces a heuristic iterative sampling process to generate diverse conformations from the predicted geometric restraints. To systematically evaluate their contributions, we conducted a series of ablation experiments, as summarized in Figure 3 and Table S3. As a point of reference, the original trX2 produced models with average RMSD values of 5.00 Å for the apo state and 4.77 Å for the holo state, setting a baseline for assessing performance improvements. Detailed definition of the ablated variants is provided in Table S3.

Building on the baseline model, we first evaluated the impact of NMR‐based fine‐tuning, which produced trX2 (NMR) (model (2) in Figure 3a) with average RMSDs of 4.94 Å (apo) and 4.17 Å (holo), both lower than trX2. This improvement suggests that fine‐tuning with NMR data provides structural diversity beyond that captured by the original trX2, thereby enhancing its ability to predict multiple conformations. Interestingly, we observed that both the fine‐tuned variant and the original trX2 networks demonstrated distinct advantages on certain samples. As illustrated in Figure S6a,b, trX2 (NMR) outperformed trX2 for nearly half of the targets (blue points; 14/37 for the apo state, 17/37 for the holo state), likely benefiting from the additional dynamic information inherent in NMR ensembles. Conversely, trX2 (NMR) performed worse than trX2 for other targets (orange points). This could be attributed to the noise introduced by the lower resolution and uncertainty associated with NMR structures, which might negatively impact the training data quality. These results highlighted a strong complementarity between trX2 and trX2 (NMR), indicating the potential of integrating both models to achieve more accurate multi‐conformation predictions.

Subsequently, we assessed the impact of the heuristic iterative sampling process. Employing this process to trX2 (i.e., model (3) in Figure 3a) reduced the average RMSD from 5.00 to 4.52 Å for the apo state, and from 4.76 to 4.16 Å for the holo state. Furthermore, applying the heuristic iterative process to trX2 (NMR) (i.e., model (4)) also reduced the RMSD from 4.94 to 4.53 Å (apo) and from 4.17 to 3.79 Å (holo). These results demonstrate the effectiveness of the sampling process for both trX2 and its NMR‐based variant, highlighting its broad applicability. These consistent performance improvements underscore the efficacy of this sampling process in exploring conformational landscapes.

Considering the complementary modeling potential of trX2 and trX2 (NMR), we analyzed the benefits of integrating predictions from both. The integration of these two models (i.e., model (5) in Figure 3a) achieved average RMSD values of 4.37 Å (apo) and 3.84 Å (holo), surpassing single‐model predictions. Building on these promising results, trX2‐D (model (6) in Figure 3) further combined the predictions from both model (3) and model (4) (i.e., the models equipped with the heuristic iterative sampling). This comprehensive integration yielded the best overall performance, with average RMSD values of 3.98 Å (apo) and 3.37 Å (holo), representing improvements of 20.4% and 29.2% compared to trX2, respectively. This strategy effectively harnesses the strengths of both models while mitigating their individual limitations.

For a more specific illustration of the distinct impacts of NMR‐based fine‐tuning and the heuristic iterative sampling process, we analyzed a challenging test case characterized by a significant inter‐domain conformation change (apo PDB ID: 1VR6, holo PDB ID: 1RZM). This protein is 3‐deoxy‐D‐arabino‐heptulosonate‐7‐phosphate synthase (DAHPS) [23], a key enzyme for aromatic amino acid biosynthesis. Its activity is regulated by an allosteric transition between inactive and active forms upon ligand binding (Cd^2^^+^, PEP, and E4P), reflected as a substantial inter‐domain motion involving a ∼160° rotation (RMSD_apo‐holo_ = 10.10 Å). This pronounced conformational difference makes DAHPS a particularly challenging and informative test case for methods aiming to capture conformational diversity.

As shown in Figure 3b, while trX2 generates a structure approximating the holo state (RMSD = 4.51 Å), it fails to accurately model the secondary structure of the variable domain in the apo state, yielding a higher RMSD of 6.17 Å. In contrast, trX2 (NMR) correctly models the secondary structure for this domain in the apo state, leading to a slight improvement in the apo prediction (RMSD = 5.97 Å). However, its performance on the holo state diminishes (RMSD = 6.37 Å), reflecting the potential negative effect associated with NMR fine‐tuning. This case further illustrates the complementarity between trX2 and trX2 (NMR). In comparison, trX2‐D demonstrates superior performance for both states. Through the heuristic sampling and multi‐model integration, trX2‐D achieves RMSD values of 4.11 Å for the apo state and 4.20 Å for the holo state, which are 33.3% and 6.8% lower than trX2, respectively. Importantly, trX2‐D effectively captures both the detailed intra‐domain secondary structures and the large‐scale inter‐domain motion.

### 2.5 Comparison with AF2‐based methods

To benchmark trX2‐D against other strategies, we further evaluated it alongside two representative AF2‐based methodologies: AF‐Cluster [15], which generates multiple conformations via DBSCAN clustering of the input MSA, and the recently published AFsample2 [24], which employs random MSA column masking to simulate the perturbation of co‐evolutionary information.

**FIGURE 4:**
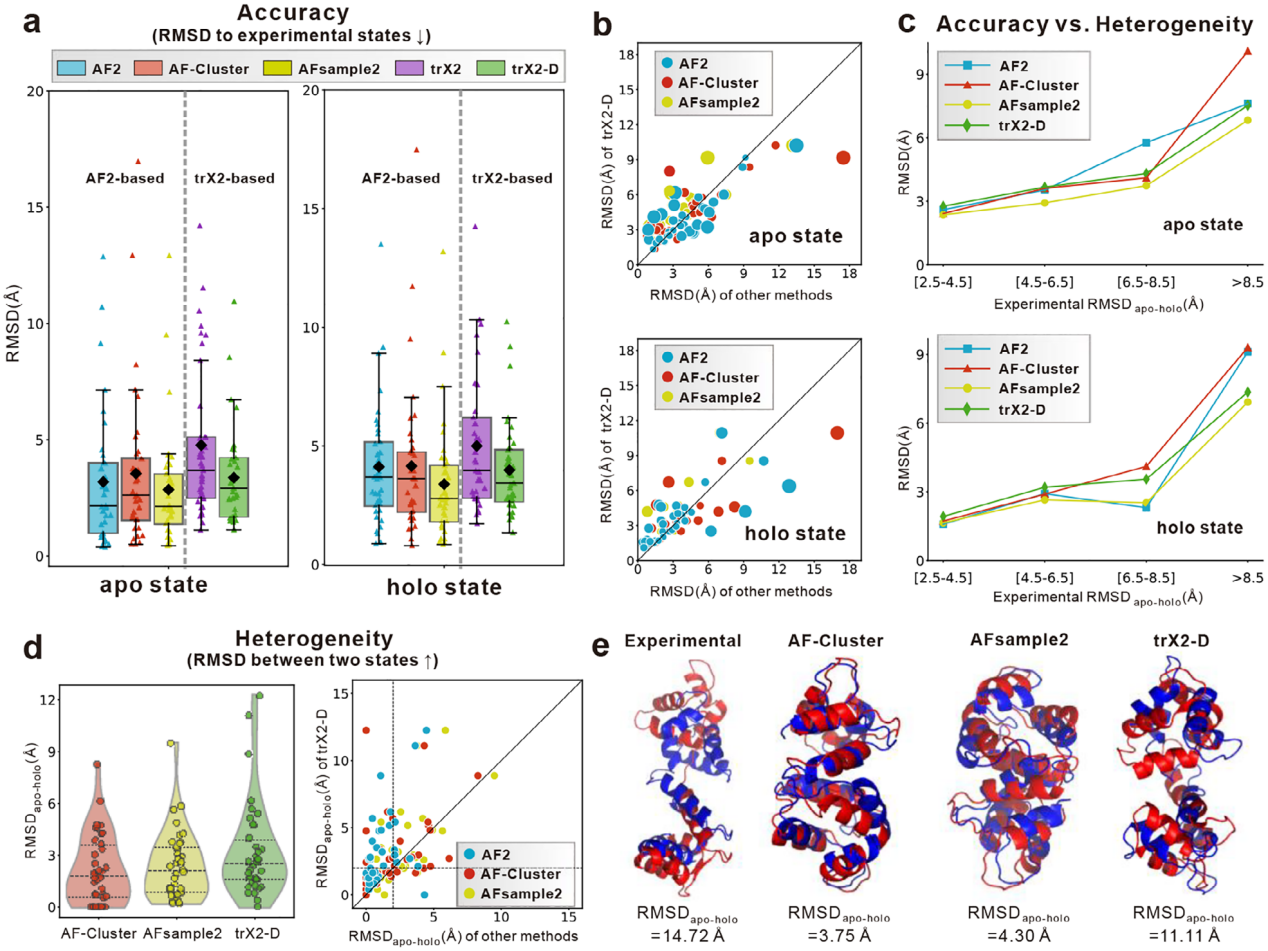
Comparison with AF2‐based methods on the apo‐holo benchmarks. (a) Box plots illustrating the RMSD distribution of predicted structures relative to experimental references for AF2, AF‐Cluster, AFsample2, trX2, and trX2‐D, stratified by apo and holo states. Black diamonds denote mean values. (b) Head‐to‐head RMSD comparison between trX2‐D and AF2‐based methods. Point sizes are proportional to the magnitude of the performance difference. (c) Correlation between prediction accuracy and the magnitude of experimental conformational change (RMSD_apo‐holo_). (d) Evaluation of conformational heterogeneity, quantified by the pairwise RMSD between the two predicted states (RMSD_apo‐holo_). In the violin plots, the internal lines represent the quartiles (dashed) and median (solid). Dashed vertical and horizontal lines in the scatter plot mark a structural similarity cutoff of 2 Å. Arrows in the titles of (a) and (d) denote the direction of better performance. Individual points in (a) and (d) represent specific targets. (e) Representative case study (EhCaBP; PDB: 1JFJ/1JFK) illustrating the superior capability of trX2‐D in capturing large‐scale conformational dynamics (apo: red; holo: blue).

To facilitate a thorough understanding of trX2‐D's capabilities, a preliminary comparative analysis between the original trX2 and AF2 was conducted on the apo‐holo dataset. As shown in Figure 4a and Table S4, the original trX2 performs worse than AF2 on both apo and holo states. This disparity can be attributed to two main factors: 1) the relatively lightweight architecture of trX2 compared to AF2 (Table S1 and Figure S1); 2) critically, the potential data leakage implied in AF2's training. The release dates for all 37 apo‐holo pairs in our dataset (all prior to 2015; Table S2) predate AF2's training data cutoff (2018‐05), suggesting AF2 might “remember” these native conformations rather than genuinely predicting them. We also find that AF2 tends to favor the holo state, with an average RMSD of 3.19 Å, compared to 4.12 Å for the apo state. For 64.9% of the 37 conformation pairs, the AF2 prediction was closer to the holo state (lower RMSD) than the apo state. This observation is consistent with findings from previous work [20]. We hypothesize that holo forms are more stable than the apo forms and are therefore more readily predicted by AF2, which excels as a well‐trained static structure prediction method.

Owing to disparate baseline performances, we focus on evaluating the performance gains yielded by each strategy over its respective baseline (AF2 or trX2). For AF‐Cluster, the MSA clustering strategy yields no statistically significant improvement over the standard AF2 baseline for either apo (P‐value: 0.53) or holo (P‐value: 0.83) state predictions. This aligns with recent findings [25], which indicate that sequence clustering strategies yield limited benefits. In contrast, trX2‐D consistently outperforms the original trX2 in predicting both states, achieving RMSD reductions of over 1 Å (P‐value: 0.0016 for the apo state and 0.0011 for the holo state). Consequently, despite using a lightweight network and avoiding AF2‐associated data leakage, trX2‐D achieved a slightly lower apo‐state RMSD and comparable overall performance. As shown in Figure 4b, trX2‐D surpassed AF‐Cluster in predicting the apo state for 51.4% (19/37) of targets, validating its competitive performance.

To gain a deeper understanding of these results, we next assessed the influence of conformational divergence on the performance comparison (Figure 4c). It has been previously observed that AF2 performs poorly for proteins exhibiting significant conformational changes [20]. For trX2‐D, we observe a tendency to outperform AF‐Cluster for proteins with large conformational changes. For example, for the targets with RMSD_apo‐holo_ over 8.5 Å, trX2‐D can generate more accurate structures for both apo and holo states, achieving RMSDs of 7.53 and 7.35 Å, respectively, compared to 7.90 and 8.64 Å of AF‐Cluster. This highlights trX2‐D's better capacity to capture significant structural variability.

We also benchmarked against the recently published AFsample2, which generates multiple conformations by randomly masking MSA columns. Unlike AF‐Cluster, AFsample2 effectively improves performance for both states over AF2, achieving the best overall performance among the compared methods, consistent with recent reports that random subsampling is superior to clustering [25]. However, in terms of relative improvements over the baseline model, trX2‐D still maintains the best performance, outperforming AFsample2 (1.02 vs 0.73 Å for apo; 1.40 vs 0.34 Å for holo). Furthermore, as shown in Figure 4d, trX2‐D is more effective at capturing conformational heterogeneity, with an average RMSD_apo‐holo_ of 3.20 Å, higher than both AFsample2 (2.38 Å) and AF‐Cluster (2.15 Å). This capability is exemplified by the EF‐hand calcium‐binding protein (EhCaBP; PDB IDs: 1JFJ/1JFK), which involves extensive inter‐domain rearrangements (RMSD_apo‐holo_ = 14.72 Å; Figure 4e). While AF‐Cluster and AFsample2 remain trapped in a single conformation, trX2‐D successfully reveals the inherent structural plasticity associated with this large‐scale transition.

To summarize, although AF2‐based methods exhibit high accuracy driven by model complexity and data leakage issues, trX2‐D yields more significant improvements over its baseline and excels in resolving conformational heterogeneity. These findings underscore the effectiveness of our output‐driven approach.

### 2.6 Comparison with Cfold to Exclude Data Leakage Bias in AF2

Current state‐of‐the‐art methods for predicting multiple protein conformations predominantly rely on the pretrained AF2 model. However, these methods are susceptible to data leakage issues when evaluating on dual‐conformation datasets, which can bias benchmark comparisons. To mitigate this bias, Cfold [10] retrained the AF2 network on a strict data split, which is designed to exclude any conformational redundancy between training and test sets.

Therefore, benchmarking against Cfold on its rigorously constructed test sets is crucial to eliminate the confounding effects of data leakage. We evaluated our method on 20 targets from this dataset, which exhibit substantial conformational changes and are non‐redundant to the training sets of trX2‐D. Each target was annotated with “Fold1” and “Fold2” states (see Methods for details on dataset construction and annotation). The information on these 20 conformation pairs is listed in Table S5.

During inference, Cfold employs two established strategies to generate multiple conformations: (1) “dropout”, which activates dropout layers during neural network inference [26]; and (2) “cluster”, which involves subsampling input MSAs at varying depths [16]. Notably, the term “cluster” here refers to the AF2 hyperparameter `max_msa_clusters` (which controls the maximum number of rows in the MSA representation), rather than the DBSCAN clustering employed in AF‐Cluster.

**FIGURE 5:**
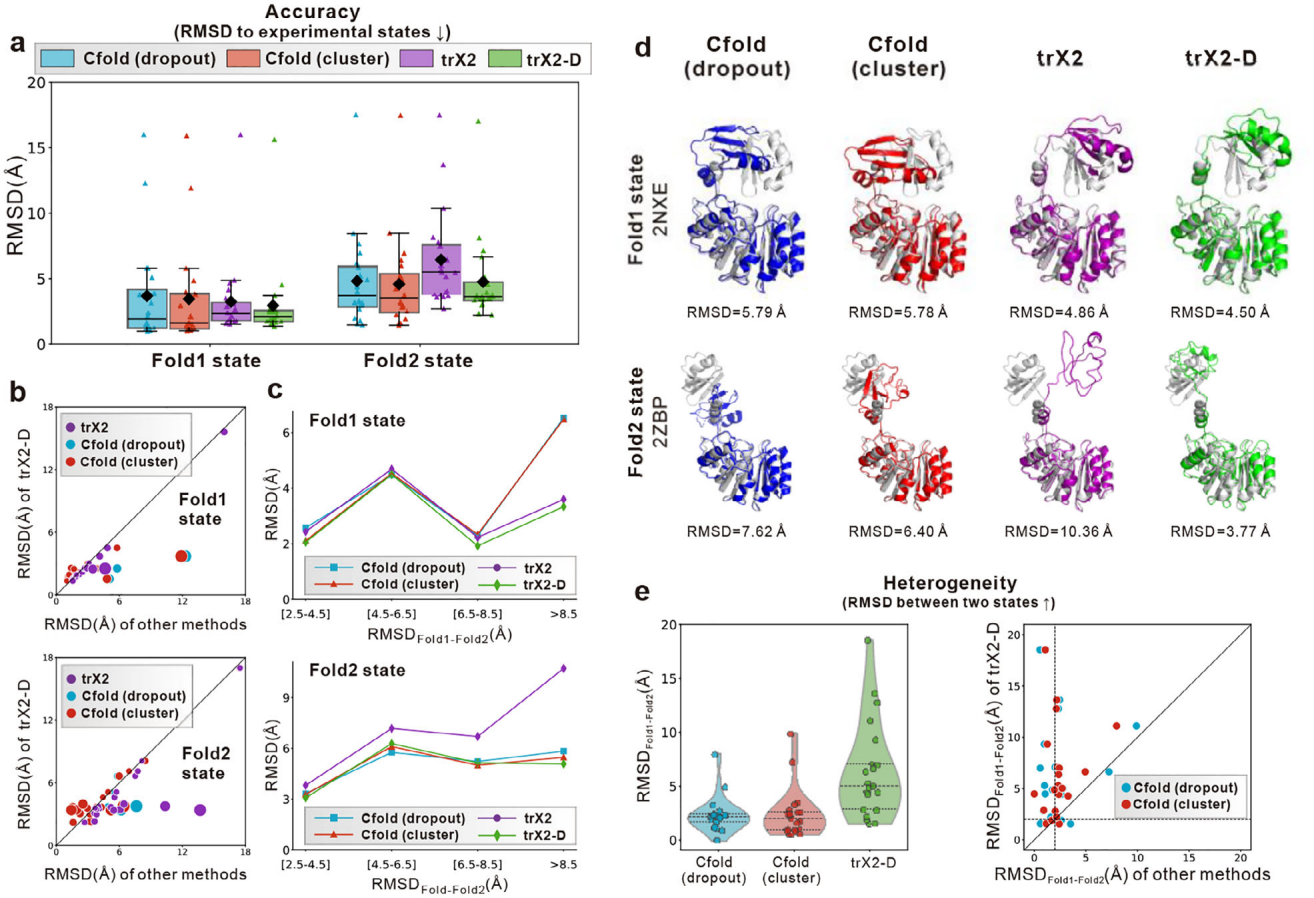
Comparison with Cfold on 20 dual‐conformation proteins from the Cfold benchmark. (a) Box plots illustrating the RMSD distribution of predicted structures relative to experimental references for Cfold, trX2, and trX2‐D, stratified by Fold1 and Fold2 states (n = 20). Black diamonds denote mean values. (b) Head‐to‐head RMSD comparison between trX2‐D and Cfold. Point sizes are proportional to the magnitude of the performance difference. (c) Correlation between prediction accuracy and the magnitude of experimental conformational change (RMSD_Fold1‐Fold2_). (d) Representative case study (ribosomal protein L11 methyltransferase; PDB: 2NKE/2ZBP) illustrating the superior capability of trX2‐D in capturing large‐scale fold switching (predicted: colored; experimental: gray). (e) Evaluation of conformational heterogeneity, quantified by the pairwise RMSD between the two predicted states (RMSD_Fold1‐Fold2_). In the violin plots, the internal lines represent the quartiles (dashed) and median (solid). Dashed vertical and horizontal lines in the scatter plot mark a structural similarity cutoff of 2 Å. Arrows in the titles of (a) and (e) denote the direction of better performance. Individual points in (a) and (e) represent specific targets.

The results on the Cfold dataset are detailed in Figure 5 and Table S6. As shown in Figure 5a, the two strategies employed by Cfold exhibit similar performance, with the “cluster” strategy slightly outperforming the “dropout” strategy, consistent with the original Cfold benchmark results [10]. Our baseline model, trX2, remains competitive with Cfold for the Fold1 state but underperforms on the Fold2 state. This highlights the gap in complexity between the baseline models, which becomes more pronounced when predicting the more challenging Fold2 state.

Despite this disparity, trX2‐D achieves improvements for both states, with gains being particularly significant for Fold2. Consequently, trX2‐D outperforms Cfold on Fold1 (RMSD: 2.95 vs 3.45 Å) and delivers competitive performance on Fold2 (RMSD: 4.75 vs 4.58 Å). Head‐to‐head comparisons in Figure 5b further confirm the competitiveness of trX2‐D. Furthermore, as illustrated in Figure 5c, trX2‐D captures large conformational changes more effectively than the compared methods (e.g., the case in Figure 5d). This finding, which aligns with results from the apo‐holo dataset, largely stems from trX2‐D's superior capability in resolving conformational transitions (Figure 5e).

Collectively, these findings demonstrate that despite the lightweight architecture of trX2, our output‐driven strategy effectively mitigates this limitation, ensuring competitive performance in rigorous benchmarks where data leakage is excluded.

### 2.7 Application to Dynamic Structures Determined by NMR Spectroscopy

The prediction of dynamic protein structures poses a more significant challenge compared to that of proteins exhibiting only two stable conformational states. To evaluate the capabilities of trX2‐D in this context, we applied it to a benchmark dataset comprising 31 proteins with dynamic structures solved by NMR spectroscopy. Evaluation was restricted to well‐restrained residues to ensure data quality (see Methods for details).

For a balanced comparison, we first quantified the ability of the predicted ensembles to cover the native ensemble using RMSD_rec_ (where “rec” refers to “recall”), defined as the average RMSD between each NMR model and its nearest neighbor in the predicted ensemble:

(1) $$\mathrm{RMS}D_{\mathrm{rec}}=\frac{1}{N}\sum_{i=1}^{N}\left[\min_{P\in P}\mathrm{RMSD}\left(S_{i},P\right)\right]$$

where *N* is the total number of NMR structure models, *S_i_* refers to the *i‐*th NMR model, P represents the set of predicted conformations, and the minimum RMSD is found by comparing *S_i_* to each prediction *P* within the set P.

**FIGURE 6:**
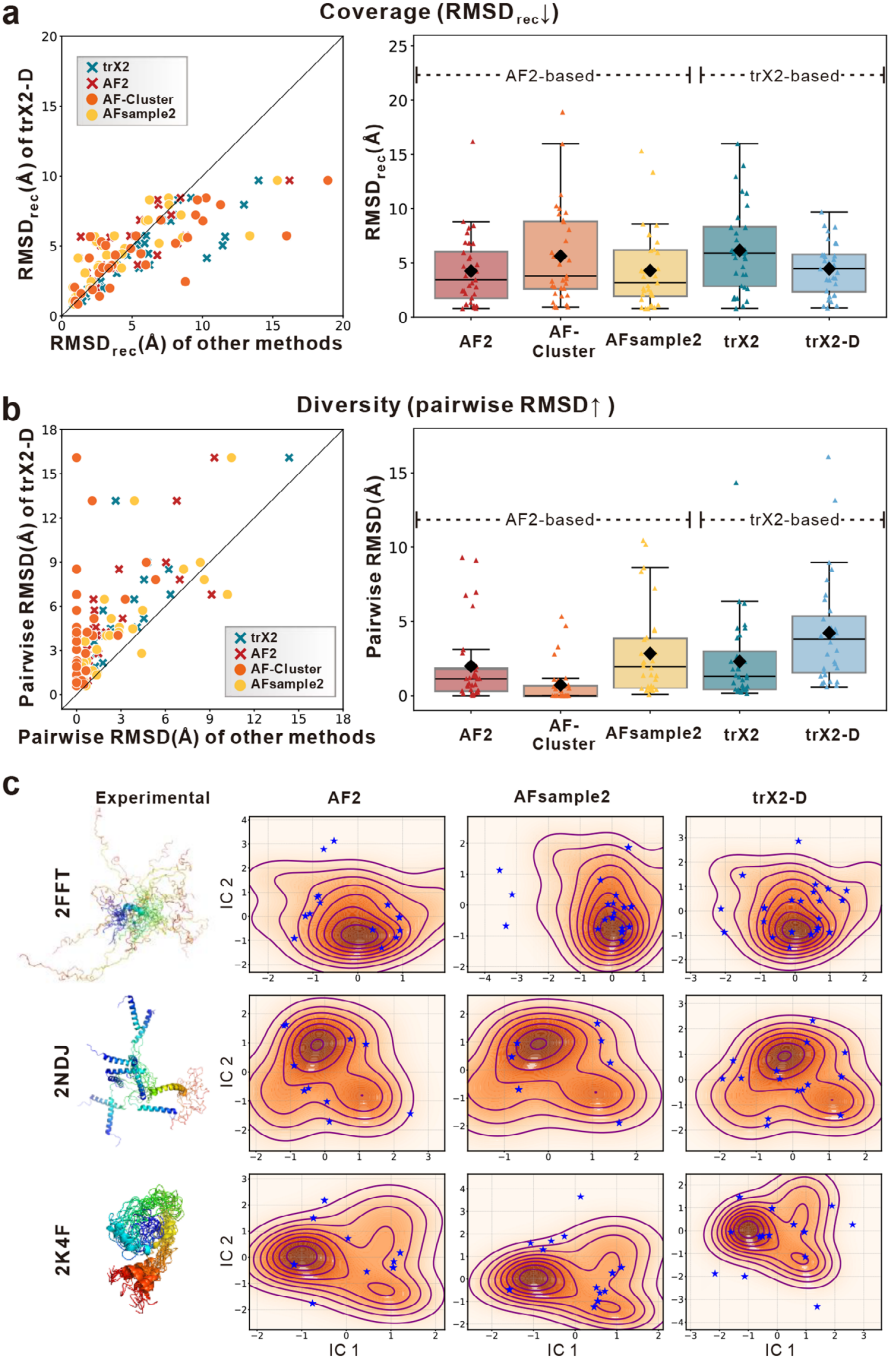
Benchmarking on the NMR dynamic protein dataset. (a, b) Comparative evaluation of ensemble generation methods across 31 NMR targets (n = 31). (a) Assessment of ensemble coverage, quantified by RMSD_rec_ (the minimum RMSD of the predicted ensemble relative to each experimental conformation). (b) Evaluation of ensemble diversity, measured by the mean pairwise RMSD within the predicted ensemble. In the box plots, black diamonds denote mean values, while individual points represent specific targets. Arrows in the panel titles denote the direction of better performance. (c) Visualization of conformational landscapes for three representative targets exhibiting significant structural heterogeneity (PDB IDs: 2FFT, 2NDJ, 2K4F). Landscapes are projected onto the first two independent components (IC1 and IC2) derived from independent component analysis (ICA) of Cα coordinates. Density contours (orange) represent the distribution of the experimental NMR ensemble, while blue stars mark the conformations predicted by each method. The corresponding experimental ensembles are depicted as rainbow‐colored cartoons on the left.

Similar to the above experiments, we compare trX2‐D with trX2 and AF‐based methods on this dynamic protein dataset. As shown in Figure 6a, trX2‐based methods generally exhibit slightly higher RMSD_rec_ values than AF2‐based methods, likely reflecting trX2's lighter architecture and potential data leakage in AF2 training, as 29 of the 31 proteins were released before the AF2 training cutoff (May 2018). However, trX2‐D demonstrates a most significant improvement over the baseline model, achieving an RMSD reduction of over 1.69 Å compared to trX2 (P‐value: 0.00019). In contrast, AF‐sample2 shows no statistically significant improvement (P‐value:0.563), while AF‐Cluster exhibits a decline in performance with an RMSD increase of 1.37 Å.

Beyond accuracy metrics, capturing the capacity to sample diverse structures is a prerequisite for characterizing protein dynamics. Evaluating ensemble diversity via mean pairwise RMSD (Figure 6b) shows that trX2‐D generates the most heterogeneous predictions (4.22 Å), significantly exceeding trX2 (2.30 Å) and AF2‐based methods (1.98–2.84 Å). Notably, AF‐Cluster fails to capture conformational heterogeneity for these targets, exhibiting the lowest pairwise RMSD (0.71 Å), even lower than the original AF2. This failure correlates with the difficulty of clustering the shallow MSAs available for these NMR proteins (Figure S7). These results highlight the unique potential of trX2‐D to explore the conformational landscape even when evolutionary information is too sparse for MSA‐based strategies.

To further assess the overall quality of the generated ensembles, we calculate RMSD_mean_, defined as the average RMSD of all predicted conformations against the average conformation derived from the NMR ensemble. As shown in Figure S8, the more diverse ensemble generated by trX2‐D does not compromise structural quality relative to the trX2 baseline. In contrast, both AF‐Cluster and AFsample2 exhibit slight degradation compared to the original AF2. These results indicate that trX2‐D effectively enhances conformational coverage and diversity without sacrificing ensemble quality.

To illustrate improved conformational sampling, we visualize the conformational spaces of the 3 samples that exhibit significant structural heterogeneity even within regions defined by sufficient experimental restraints (Figure 6c). Intriguingly, we observed that the structural heterogeneity in these cases often involves intrinsically disordered regions (IDRs) or highly flexible loops. These regions pose a considerable challenge for AF2 and AFsample2, which are primarily trained on stable, well‐ordered structures and thus tend to underestimate dynamics. Consequently, predictions from AF2‐based methods are often confined to narrow basins in the independent component analysis (ICA) projections (Figure 6c), covering only a limited spectrum of the experimental conformational landscapes. In contrast, trX2‐D generates a more diverse set of predictions that span a broader conformational range. This enables trX2‐D to more effectively capture the structural flexibility inherent in these disordered regions, and consequently yields more diverse ensembles than the AF2‐based approaches (as evidenced by the higher pairwise RMSD). This observation is reinforced by per‐residue root mean square fluctuation (RMSF) analysis restricted to well‐restrained residues (Figure S9). For these highly flexible samples, AF2 and AFsample2 underestimated structural fluctuations compared to the NMR ensemble, whereas the fluctuations captured by trX2‐D models closely correspond with those of the NMR states, reflecting a more accurate depiction of protein flexibility.

We further assessed the agreement between predicted ensembles and NMR observables. First, we analyzed backbone flexibility using ensemble‐derived [27] generalized order parameters [28, 29] (S^2^_ensemble_), which quantify the spatial restriction of bond vector motions (see Methods for details). We evaluated the Pearson correlation (r) between the predicted and reference (NMR) profiles, focusing exclusively on well‐restrained residues. As shown in Figure S10, trX2‐D exhibits the highest median correlation (0.504) with the reference NMR ensembles, significantly higher than AF2‐based methods (0.076‐0.201). This demonstrates the superior capability of trX2‐D in capturing the correct backbone dynamics.

Second, we evaluated global structural orientation using residual dipolar coupling [30] (RDC). Unlike local distance constraints, RDCs capture the orientation of specific chemical bonds with respect to a global alignment frame, thereby offering a rigorous metric for overall structural topology. The agreement with experimental data was quantified by the Q‐factor [31] (lower is better, see Methods). Among the 31 NMR targets, only two possess high‐quality experimental data suitable for this analysis (PDB IDs: 2M3E and 2M6M). For 2M3E, trX2‐D achieves the lowest Q‐factor (0.12), superior to trX2 (0.35) and AF2‐based methods (0.90–1.35) (Figure S11), confirming that our ensemble captures realistic orientational dynamics rather than stochastic noise. However, for target 2M6M, all methods exhibit significant deviation from the experimental values (Q‐factors > 0.6). As shown in Figure S11c, while these methods roughly identify the correct flexible regions, they fail to capture the precise spatial orientation of these fluctuations relative to the core, leading to the observed discrepancy with the experimental RDC profile. This highlights the persistent challenge of accurately modeling the directionality of structural dynamics in many cases.

Capitalizing on the unique advantages of NMR in capturing protein dynamics, future improvements may involve incorporating NMR data and restraints directly into model training [32]. This approach would ensure that the network learns genuine biological dynamics rather than fitting to artificial noise.

## 3 Discussion

Structural heterogeneity underpins protein function [33], yet predicting multiple protein conformational states remains a formidable challenge. Current mainstream methods primarily utilize the multi‐conformational information embedded in MSA (i.e., input‐driven strategies) [10, 15, 16]. In contrast, earlier studies have shown that *de novo*‐predicted contact maps obtained through deep learning often contain structural information about multiple states [13]. Motivated by this insight, we observed that the predicted 2D geometries also encode such information, as revealed by their multi‐peaked distributions (Figure S15). However, a systematic approach to utilize 2D geometries for predicting multiple conformations has remained unexplored.

Building upon this observation, we introduce trX2‐D, an automated approach for predicting alternative protein conformations by employing a heuristic iterative sampling process on the 2D geometries predicted by trX2. Table S7 provides a specific contrast between trX2‐D and input‐driven strategies, detailing their respective mechanisms, advantages, and limitations. In contrast to existing input‐based methods, trX2‐D, as an output‐driven sampling method, is capable of generating more diverse conformations even without prior knowledge of specific structural states, as its sampling is directly performed on the *de novo*‐predicted structural restraints. We have rigorously assessed trX2‐D with three independent datasets, including two dual‐conformation sets and one dynamic structure set. Benchmarking results demonstrate that trX2‐D significantly outperforms trX2 in predicting alternative conformations. Notably, when evaluating the magnitude of improvement relative to the respective baselines, trX2‐D outperforms AF2‐based methods (AF‐Cluster and AFsample2), despite the inherent architectural gap between trX2 and AF2. By modulating predicted geometry rather than sequence inputs, trX2‐D successfully captures broader conformational heterogeneity that is often inaccessible to MSA‐based strategies. This independence from evolutionary depth also positions trX2‐D as a promising tool for challenging systems, such as orphan proteins lacking sufficient sequence homologs.

Moreover, our output‐driven strategy offers a unique advantage: interpretability. The use of energy‐based sampling facilitates the construction of an energy landscape, exemplified by the classic Adenylate Kinase (AdK) case, where energy variations physically elucidate the conformational switching pathway. Furthermore, physical energy scoring enables the exclusion of energetically unfavorable conformations. For instance, filtering out structures with outlier Rosetta energies (see Methods) slightly improves ensemble quality for nearly all targets (90.3%, 28/31) in the NMR benchmark, with minimal compromise to accuracy (Figure S12). Although CPU‐based minimization currently results in longer inference times (Table S8), this represents a technical rather than a conceptual bottleneck. This limitation is addressable by transitioning from the PyRosetta framework to GPU‐accelerated frameworks, such as integrating high‐performance simulation backends or differentiable force fields [34, 35].

trX2‐D's slightly lower accuracy compared to AF‐Cluster reflects the architectural gap between trX2 and AF2 (Figure S1) and AF2's data leakage (Tables S2 and S8), rather than methodological inferiority. We addressed the leakage concern in the Results section by benchmarking against Cfold, which represents an AF2 variant free from data leakage bias. Moreover, to rigorously compare the sampling strategies themselves, we implemented MSA clustering on trX2 (trX2‐Cluster). Notably, trX2‐D outperforms trX2‐Cluster across all states (Figure S13), validating that our heuristic iterative strategy is more effective than MSA clustering within the trX2 framework.

Subsequently, we explore applying our heuristic iterative strategy to AF2‐predicted distance maps (AF‐HIS), but observe no improvement over the standard, end‐to‐end AF2 (Figure S14a). This is likely due to the superiority of the AF2 structure module over PyRosetta minimization and the lack of dynamic signals in AF2's sharp, unimodal distance distributions (Figure S15). Inspired by findings that shallow MSAs may induce multi‐conformational signals in AF2 [15, 16], we filter each MSA using HHfilter [36, 37] to retain only 10 representative sequences. While this slightly increased the diversity of AF2 distance distributions (AF2 (shallow) in Figure S15), it still fell short of the diversity observed in trX2. To decouple the sampling effect from the structure module's bias, we performed energy minimization based on AF2‐predicted distances (AF‐Rosetta). Using shallow MSAs, AF‐HIS improved upon the holo‐preferred AF‐Rosetta in predicting the apo state for 70.2% (26/37) of cases (Figure S16b), particularly for targets with large conformational changes (Figure S16c). However, the gains were smaller than those with trX2, highlighting a limitation of geometry‐based sampling: it depends on multi‐state signals in 2D geometries and struggles when predictions are highly confident in a single state.

Another challenge, which is common for all the existing multi‐conformation prediction methods, is the automated and efficient selection of biologically meaningful conformations from the generated ensembles. While trX2‐D excels at generating diverse conformational ensembles, it still faces the challenge of selecting representative conformations, especially for dual‐conformation proteins. Preliminary efforts using k‐means clustering based on standard structural similarity metrics (TM‐score, RMSD, and inter‐Cα distances [38]) proved insufficient, as illustrated by the 0.2∼0.3 Å higher RMSDs in average after clustering (Figure S17). This indicates a promising future direction for advancing multi‐state structure prediction, that is, exploring more sophisticated conformation selection/clustering strategies to better identify biologically meaningful conformations from generated structures. Notably, the efficacy of energy‐based filtering (Figure S12) underscores that incorporating broader physical, experimental, and biological constraints, such as experimental B‐factors and functional site conservation, represents a promising avenue for future improvements.

Conformation generation in trX2‐D is primarily powered by energy minimization, which involves both predicted 2D geometries and physical energy terms from Rosetta. Recently, generation models, especially the diffusion models, have shown promise in protein structure generation [39, 40, 41, 42]. However, due to the lack of physical restraints, these generative models alone may struggle to generate conformations that obey the Boltzmann distribution. While several methods (e.g., CONFDIFF [43], DiG [44]) have made strides in incorporating physical guidance into diffusion and/or sampling procedures, accurately defining force fields and efficiently selecting biologically meaningful conformations continue to be major challenges. The path forward will likely involve a more sophisticated integration of deep generative models with physical/biological restraints, not only to improve the effectiveness of generating diverse conformations but also to better capture those allosteric transitions critical for protein function [32]. Furthermore, extending predictions from equilibrium states to dynamic folding pathways has emerged as a promising frontier, capturing growing attention in the field [45, 46].

## 4 Methods

### 4.1 Construction of Datasets

We constructed three benchmark datasets and two training sets, with rigorous filtering to ensure no redundancy between training and testing (Table S10).

#### 4.1.1 Test sets

Three benchmark datasets were constructed in this work. The first dataset consists of 37 apo‐holo protein pairs collected from a recent work by Saldano et al., [20]. From their original set of 91 pairs, 87 with identical sequences between the apo and holo states were initially selected. To focus on pairs with substantial conformational differences, we only retained the 37 protein pairs with significant conformational change, defined as having a TM‐score_apo‐holo_ value below 0.8 or an RMSD_apo‐holo_ value above 6 Å. While a TM‐score_apo‐holo_ of 0.8 was frequently used as the cutoff to identify significant conformational changes [10, 13, 17], our analysis revealed cases where high TM‐scores between states can also coincide with substantial structural differences, as indicated by RMSD_apo‐holo_ values exceeding 6 Å (Figure S18). For example, for the DAHPS enzyme, the transition between states involves significant interdomain variation (apo PDB ID: 1RZM, holo PDB ID: 1VR6; Figure S18a). However, its TM‐score_apo‐holo_ value was over the 0.8 cutoff. Therefore, to ensure a robust evaluation, we incorporated such cases into our benchmark set.

The second dataset was obtained from the Cfold benchmark set [10], which includes 243 dual‐conformation proteins with pairwise TM‐score<0.8. For consistency, we only considered 155 samples for which Cfold provided the predicted structures in its Zenodo repository (https://zenodo.org/records/10837082). To prevent data leakage, we further filtered this dataset using the cd‐hit [47, 48] (V4.8.1) program at a 40% sequence identity threshold relative to our training set. After this step, we removed samples with sequence lengths >300 to save computational time, resulting in 20 unique samples. For confirmation annotation, we categorized proteins based on the RMSD of their AF2 predictions. For each conformation pair, the conformation with lower RMSD in at least 3 out of 5 AF2 predictions was labeled as “Fold1”, while the other conformation in the pair was designated as “Fold2” [17].

The third dataset was derived from the 292 dynamic proteins identified by NMR spectroscopy, reserved from the dataset used to fine‐tune trX2 (see below), each protein with an average of 19 NMR models. The proteins sharing over 30% sequence identity relative to all the training sets were excluded, resulting in 118 samples. To ensure initial conformational diversity, only ensembles with a minimum pairwise TM‐score below 0.8 or a maximum pairwise RMSD over 6 Å were retained, resulting in 92 proteins. Subsequently, 29 proteins were excluded due to insufficient MSA depth (<10) for running AF‐Cluster. Furthermore, one protein (PDB ID: 6XRY) with extreme conformational dynamics (maximum pairwise RMSD = 33.9 Å) was also excluded, as all evaluated methods failed to generate structure ensembles for this target, resulting in an intermediate set of 62 proteins.

To ensure our analysis was rigorously validated against experimental evidence, we further restricted the dataset to 44 targets with available NMR restraint files. For these proteins, we focused our analysis exclusively on residues within well‐defined core regions, defined as those supported by at least two non‐sequential Nuclear Overhauser Effect (NOE) restraints (sequence separation ∣*i* − *j*∣ ≥ 2). Finally, we re‐evaluated the conformational diversity within these core regions using the aforementioned thresholds (TM‐score < 0.8 or RMSD > 6 Å), yielding a final curated dataset of 31 proteins with significant conformational dynamics.

Duplicate samples across the above three datasets were removed to eliminate redundancy. The final datasets consist of 37 apo‐holo proteins, 20 two‐state proteins, and 31 dynamic proteins, respectively.

#### 4.1.2 X‐Ray Training Set

This training set was derived from the 15 051 X‐ray protein chains collected in trRosetta [5]. These proteins were non‐redundant (sequence identity <30%), released before 2018‐05‐01 in the PDB database, resolved by X‐ray crystallography, and have pre‐constructed MSAs with at least 100 homologous sequences. To prevent data leakage during benchmark evaluations, any training set chains sharing >50% sequence identity with proteins in the benchmark test set were removed, resulting in a final training set of 14 275 chains.

#### 4.1.3 NMR Training Set

This training set was derived from a dataset of 8038 monomeric proteins with experimentally determined dynamic structures from NMR spectroscopy. A two‐stage filtering procedure was employed to ensure non‐redundancy and prevent data leakage. First, the chains were clustered at 60% sequence identity using CD‐HIT. 95% of the resulting 4746 clusters (7454 chains) were randomly selected for the initial training set, while the remaining 5% of clusters (292 chains) were reserved for the test set construction. Second, to further prevent data leakage during benchmark evaluations, any training chains sharing >50% sequence identity with proteins in the benchmark test set were removed, resulting in a final training set of 7269 chains.

### 4.2 Experimental Setup

#### 4.2.1 MSA Generation

MSAs for all proteins were generated using MMseqs2 [49] (v13.45111) by searching the UniRef50 database (E‐value threshold: 0.001; maximum 20 000 target sequences per query). Unless otherwise specified, these MSAs served as the common input for all structure prediction methods to ensure fair comparison.

#### 4.2.2 Compared Methods

We compared trX2‐D to the following structure prediction methods:

##### 4.2.2.1 trRosettaX2 (trX2)

The trX2 protocol was used to predict 2D geometric constraints from the input MSA, which guided structure folding via energy minimization in Rosetta. To sample diverse conformations, rather than relying on a fixed end‐to‐end prediction, we executed 50 independent energy minimization processes for each target. These processes incorporated randomness in both their initialization and optimization steps, ultimately yielding an ensemble of 50 structures.

##### 4.2.2.2 AlphaFold2 (AF2)

The standard AlphaFold2 without structural templates was used to generate predictions. For each target, an ensemble of 50 models was produced using distinct random seeds.

##### 4.2.2.3 AF‐Cluster

Following the AF‐Cluster pipeline, each target's MSA was clustered using the DBSCAN algorithm with default configurations, yielding 2∼566 sub‐MSAs per target. AlphaFold2 (without templates) was run on each sub‐MSA individually, producing one structure per sub‐MSA and thus a total ensemble of 2∼566 structures per target.

##### 4.2.2.4 AFsample2

We utilized the official AFsample2 implementation with default settings to generate 100 conformations. Specifically, predictions were derived from 100 randomly subsampled MSAs (key parameters: –msa_rand_fraction 0.20, –nstruct 100).

##### 4.2.2.5 Cfold

The Cfold predictions were obtained directly from the published dataset on the Zenodo repository (https://zenodo.org/records/10837082). We specifically used structures generated by Cfold's MSA clustering strategy, which was reported to outperform the alternative dropout strategy in the original study.

#### 4.2.3 Evaluation Metric

The accuracy of the predicted models was evaluated by RMSD. For each experimentally determined conformation of a target, RMSD values comparing all generated structures against this conformation were computed utilizing the TM‐score [50] program. Then the minimum RMSD value was selected to represent the accuracy for that specific conformation.

### 4.3 Order Parameter Analysis

While generalized order parameters (*S* ^2^) typically characterize time‐dependent fluctuations derived from molecular dynamics trajectories, they can also be adapted to quantify the spatial restriction of bond vectors within a conformational ensemble [27]. Here, we calculated ensemble‐derived generalized order parameters (*S* ^2^_ensemble_) to estimate the backbone flexibility of each ensemble. Since the definition of N–H bond vectors requires explicit protons, missing hydrogens in AF2‐based structures were reconstructed using PDBFixer [51] (https://github.com/openmm/pdbfixer), whereas the experimental and trX2‐based structures were processed with their existing explicit hydrogens. The order parameter for residue *j* was then computed as the squared norm of the ensemble‐averaged unit vector:

(2) $$S_{ensemble,j}^{2}=∥\frac{1}{N}\sum_{n=1}^{N}{\vec{\mu }}_{j,n}{∥}^{2}$$

where 𝑁 was the ensemble size and ${\vec{\mu }}_{j,n}$ represents the N–H unit vector of the *n*‐th conformer. To quantify predictive accuracy, we calculated the Pearson correlation coefficient (*r*) between predicted and reference profiles, restricting the analysis to "well‐restrained" residues (as defined in the "Construction of datasets" section). Notably, AF‐Cluster could not be evaluated for 14 targets because its predictions collapsed into single conformations; this resulted in uniform *S* ^2^_ensemble_ values of 1.0 across all residues (zero variance), precluding the calculation of Pearson correlation coefficients.

### 4.4 Residual Dipolar Coupling (RDC) Analysis

Experimental ^1^H–^1^ ^5^N RDC data were available for four proteins in the NMR test set. Calculating RDCs for predicted conformations requires a reference alignment tensor derived from the deposited NMR ensemble, which characterizes the global orientation of the molecule relative to the alignment medium. For the evaluation of predicted structures, we employed an ensemble‐based fitting protocol using the calcTensor module in Xplor‐NIH [52, 53]. The alignment tensor was then fitted directly to this predicted ensemble using the “‐ensemble” argument. Subsequently, this alignment tensor was applied to the predicted ensembles to calculate RDCs [54].

For each predicted ensemble, the agreement with experimental data was quantified via the Q‐factor [31], defined as the root‐mean‐square (RMS) deviation between the two RDC profiles, normalized by the RMS of the experimental values. To ensure the reliability of the analysis, we excluded targets where the Pearson correlation (*r*) between experimental and back‐calculated RDCs (for the reference structure) was less than 0.8, due to potential artifacts from internal dynamics [55]. This yielded a final dataset of two proteins (PDB IDs: 2M6M and 2M3E).

### 4.5 NMR Fine‐Tuning of trRosettaX2

To improve the ability to capture the conformational changes, we fine‐tuned the pretrained trX2 (described in Text S1) on the dynamic structures from the NMR training set. The loss function was adapted to consider all the conformations of each sample. Specifically, for each protein, we computed the loss function between the predicted structure and all native conformations and selected the minimum loss for backpropagation. This process can be written as:

(3) $$L_{\mathrm{NMR}}=\underset{Y\in S_{\mathrm{Conformation}}}{min}\frac{1}{4N^{2}}\sum_{i=1}^{N}\sum_{j=1}^{N}\sum_{g\in \left\{2D\;\mathrm{geometries}\right\}}CE\left(P_{g}\left(i,j\right),Y_{g}\left(i,j\right)\right)$$

where *S*_Conformation_ refers to the set of native conformations; *N* was the number of residues; *CE*() was the cross‐entropy function; *P_g_* (*i*,*j*) was the predicted probability distribution for the 2D geometry *g* between residues *i* and *j*; *Y_g_* (*i*,*j*) *was* the corresponding ground truth one‐hot encoding derived from the native conformation *Y*.

Throughout the training, we used the Adam optimizer with a learning rate of 0.0001 to minimize the loss function ℒ_NMR_.

### 4.6 Heuristic Iterative Process for trX2‐D

The heuristic iterative process in trX2‐D was designed to generate a diverse ensemble of protein conformations from the 2D geometries predicted by trX2 and trX2 (NMR). At each iteration, geometry information from the prior iteration's 3D conformation was selectively excluded from the current 2D geometries. These updated 2D geometries were subsequently used to generate a new conformation through energy minimization. Sufficient iterations of this process can yield a diverse conformational set. This procedure is illustrated in Figure 1C and Figure S2.

Formally, let $G_{n}$ denote the set of 2D geometries (1 distance + 3 orientations) at the *n*‐th iteration. *S_n_* was the corresponding 3D structure generated through energy minimization based on $G_{n}$. The *n*‐th iteration aims to update $\;G_{n}$ and *S_n_* to $G_{n+1}$ and *S*_*n* + 1_, respectively. Once the iteration process terminates, all generated structures, {*S_n_*}, were collected to form the predicted ensemble of conformations.

For convenience, let $\;p_{n}\in G_{n}$ represent the probability distribution of one of four defined geometries for a specific pair of residues. A sharp and unimodal *p_n_* signifies a highly stable geometric relationship between the corresponding residue pair, while a broad or bimodal *p_n_* may imply variability for this residue pair. Based on this hypothesis, we design a decay‐and‐smooth procedure to update *p_n_* to *p*_*n* + 1_ (Figure S2), which is detailed as follows (‖ · ‖_∞_ refers to the *L*_∞_ norm):

1. if ‖*p_n_* ‖_∞_ <  0.5, indicating potential conformational variation at this residue pair, *p_n_* will be decayed based on the geometry value calculated for the corresponding residue pair in the 3D structure *S_n_*. This operation was intended to remove the information inherent in the previously generated 3D conformation and to focus on the alternative conformation information implied in the remaining distribution regions. The decayed distribution was then normalized and smoothed with a Gaussian filter to ensure structural regularity during energy minimization (see Figure S19 for an example).
2. if ‖*p_n_* ‖_∞_ ≥  0.5, indicating this residue pair was highly stable, *p_n_* will remain unchanged.

In total, the update rule is defined as:

(4) $$p_{n+1}=\left\{\begin{array}{cc} \left(f\circ g\right)\left(p_{n}-0.5p_{n}⊙p_{n}^{s}\right), & |\left|p_{n}\right|{|}_{\infty }<0.5\\ p_{n}, & |\left|p_{n}\right|{|}_{\infty }\geq 0.5 \end{array}\right.$$

where ⊙ denotes element‐wise multiplication, *g* represents normalization, and *f* denotes Gaussian smoothing. $p_{n}^{s}$ refers to the distribution (i.e., one‐hot coding) calculated from the 3D structure *S_n_*.

The updated 2D geometries $G_{n+1}$ were obtained by updating all four types of geometry across all residue pairs in the protein. These updated geometries were then used to generate a new conformation *S*_*n* + 1_. The iterative process terminates when the distributions for all residue pairs have converged (change < 0.01). To exclude energetically unfavorable conformations, we filter out structures with energy values exceeding the 75th percentile plus the interquartile range (IQR).

### 4.7 Implementation of Energy Minimization

Following the trRosetta protocol [5], calculations were performed using PyRosetta (version 2024.39+release.59628fb). A two‐stage protocol comprising coarse‐grained (centroid) minimization and full‐atom refinement was implemented. First, centroid models were optimized using the quasi‐Newton MinMover (L‐BFGS algorithm, lbfgs_armijo_nonmonotone) with a maximum of 1000 iterations and a convergence tolerance of 10^−4^. The scoring function integrated predicted restraints with standard centroid terms using the following weights: AtomPair (5.0), Dihedral (4.0), Angle (4.0), Ramachandran preference (rama, 1.0), omega torsion (omega, 0.5), steric repulsion (vdw, 1.0), and backbone hydrogen bonding (cen_hb, 1.0). Subsequently, models were converted to full‐atom representations and refined using FastRelax with the ref2015 scoring function. During relaxation, restraint weights were adjusted to AtomPair (4.0), Dihedral (1.0), and Angle (1.0).

### 4.8 Statistical Analysis

Data were presented as data points or distributions. The sample size (n) for each analysis was specified in the corresponding paragraph. One‐sided Student's *t*‐tests were employed to assess the statistical significance of performance improvements. Significance was defined as P‐value<0.05, and specific P‐values were reported in the corresponding paragraph. All statistical analyses were performed using Python 3.10 (utilizing the Pandas, SciPy, and NumPy libraries).

## Author Contributions

J.Y. conceptualized and administered the study. C.X. curated benchmark data, implemented the NMR fine‐tuning, and developed the iterative sampling procedure. W.W. designed and implemented the neural network. C.X. and W.W. conducted formal analysis. Z.P. and W.W. co‐supervised the study. All authors wrote and revised the manuscript.

## Conflicts of Interest

The authors declare no conflicts of interest.

## Supporting information




**Supporting File 1**: advs73981‐sup‐0001‐SuppMat.pdf.

**Supporting File 2**: advs73981‐sup‐0002.xlsx.

**Supporting File 3**: advs73981‐sup‐0003.xml.

## Data Availability

The web server of trX2 is available at: https://yanglab.qd.sdu.edu.cn/trRosetta/. The source codes for trX2‐D are available at: https://github.com/YangLab‐SDU/trRosettaX2‐Dynamics. The three benchmark datasets for trX2‐D are available at https://yanglab.qd.sdu.edu.cn/trRosetta/benchmark_dynamics/.
