# Supplementary material for: Generating Dynamic Structures Through Physics‐Based Sampling of Predicted Inter‐Residue Geometries
Source: Adv Sci (Weinh). 2026 Jan 21;13(18):e18469. doi: 10.1002/advs.202518469 (PMC13042367; doi:10.1002/advs.202518469)
Supplement: Supplementary file 1 — Supporting File 1: advs73981‐sup‐0001‐SuppMat.pdf. [file ADVS-13-e18469-s003.pdf]

Supplementary Materials

Supplement Text 1. Performance of trRosettaX2 on protein static structure prediction

Architecture of trRosettaX2. The trRosettaX2 architecture is primarily inspired by AlphaFold2 (AF2), but several modifications were made to optimize performance under limited computational resources, which is summarized in Table S1. First, we introduced an MSA-based language model ESM-MSA [1], which was pretrained on the MSAs of UniRef50 sequences, to generate an initial MSA embedding and 2D attention maps. Second, to accommodate the constraints of GPU memory (A100 40GB), we utilize Direct Coupling Analysis (DCA) scores for embedding the "extra MSA" rather than relying on transformer blocks. Third, observing that the pure transformer network struggles to converge with a "batch size=1" configuration, we introduced the multi-scale convolutional neural network Res2Net (R2N) [2] to assist attention-based triangle updates in the Evoformer. This modified module, termed trFormer (Fig. S1a), combines the strengths of transformers and convolutional networks—leveraging the former for global topology modeling and the latter for capturing local details. Notably, we only use 12 trFormer blocks, a significant reduction from the 48 Evoformer blocks used in AlphaFold2.

During inference, the input MSA is transformed into two representations: the MSA representation (i.e., the MSA embedding generated by ESM-MSA) and the pair representation (including the direct couplings derived from MSA and the attention maps from ESM-MSA). These two representations are then iteratively updated by 12 trFormer blocks. The first row of the updated MSA representation, along with the full pair representation, is fed into the structure module to predict the full-atom 3D structures.

The loss function of trRosettaX2 consists of several independent items, including the frame-aligned-point-error (FAPE) loss $\mathcal{L}_{FAPE}$, the 2D geometries loss $\mathcal{L}_{2D}$, the torsion loss $\mathcal{L}_{torsion}$, the pLDDT loss $\mathcal{L}_{pLDDT}$, the clash loss $\mathcal{L}_{clash}$, and the bond loss $\mathcal{L}_{bond}$. In total, the loss function can be written as:

$$\mathcal{L}_{\text{trX2}} = \mathcal{L}_{\text{FAPE}} + 0.5\mathcal{L}_{\text{2D}} + \mathcal{L}_{\text{torsion}} + 0.05\mathcal{L}_{\text{plddt}} + 0.1\mathcal{L}_{\text{clash}} + 0.2\mathcal{L}_{\text{bond}} \quad \text{(S1)}$$

The training of trRosettaX2 is performed on a single A100 40GB GPU and takes around 10 days, using the training set consisting of 14,275 protein crystal structures introduced in Methods.

Performance of trRosettaX2 for static structure prediction. To validate the effectiveness of the above modifications, we trained and evaluated several model variants on 91 domains from the CASP14 experiments. The evaluation is based on the average TM-score on the CASP14 dataset, using trRosettaX_FM (trX_FM; the template-free version of trRosettaX), RoseTTAFold (RF; the pyRosetta version), and AlphaFold2 (AF2) as reference models. During prediction, all models use the same MSA as input and do not rely on structural templates.

According to Fig. S1b, directly adopting the Evoformer architecture performs poorly, with an average TM-score of 0.576 on the CASP14 domains, which is even lower than the Res2Net-based trX_FM. This reflects the incompatibility between pure transformer networks and the training configuration we employed under limited resources (e.g., batch size of 1 and only 12 blocks). However, by incorporating Res2Net with the transformer (i.e., trFormer), the model's average TM-score significantly improves by 27.5%, surpassing trX_FM by 11.6%. Training with binary-sub-sampling[3] further increases the TM-score by approximately 2.5%, outperforming RF. With the introduction of ESM-MSA representations, the TM-score improves again by about 4%. Finally, using the same input (i.e., MSA), trX2 achieved an average TM-score of 0.781, 18.7% higher than trX_FM and 5.8% higher accuracy than RF. Additionally, the trX2 performance is competitive with AF2 (TM-score 0.781 vs 0.843), despite using fewer parameters (0.3B vs 0.9B) and computational resources (1 A100 GPU vs 128 TPUv3 cores). These results illustrate the effectiveness of the modification to accommodate the limited computational resources. On the 94 domains from CASP15, we can also observe a similar comparison (Fig. S1c). trX2 represents a good trade-off of modeling accuracy and parameters/training cost compared to other state-of-the-art methods, further demonstrating its efficiency as a static structure prediction method.

Table S1 | Methodological comparison between trX2, AF2, and RF.

Methods	Approach	Neural network architecture	Number of parameters	Training resources	Training time
trRosettaX2	End-to-end/two-steps	R2N-enhanced transformer (trFormer, 12 blocks)	30 M	1 A100 40GB GPU	~ 10 days
AlphaFold2	End-to-end	Evoformer (48 blocks) +structure module (8 blocks)	93 M	128 TPU v3 cores	~ 2 weeks
RoseTTAFold (pyRosetta)	Two-steps	3-track transformer (13 blocks)	130 M	8 V100 32GB GPUs	~ 4 weeks
RoseTTAFold (e2e)	End-to-end	3-track transformer (13 blocks) +SE3-transformer (2 layers)	137 M		

Table S2 | Information of the apo-holo proteins, along with RMSD and TM-score between apo and holo states.

Apo state		Holo state		RMSD(Å) between apo and holo	TM-score between apo and holo	trX2-D sample number
PDB ID	Release date	PDB ID	Release date			
2LAO	1993-02-25	1LAH	1993-10-06	4.75	0.70	325
1NTR	1994-09-16	1KRX	2002-01-10	4.55	0.63	276
1CFC	1995-08-02	5DOW	2015-09-11	9.25	0.38	291
1MUT	1995-09-14	1PUN	2003-06-25	4.98	0.58	212
1LIP	1995-09-21	1JTB	1996-12-03	3.29	0.66	162
4AKE	1995-12-29	2ECK	1996-12-16	6.95	0.69	306
1SYM	1996-05-29	1XYD	2004-11-09	6.69	0.54	204
1AEL	1996-07-30	1URE	1996-06-17	2.69	0.77	230
2CJO	1997-02-06	1ROE	1995-11-24	4.55	0.58	216
2RCS	1997-05-14	1AJ7	1997-05-15	7.21	0.59	351
1URP	1998-04-03	2DRI	1994-09-23	4.20	0.72	281
1SKT	1998-04-06	1TNQ	1995-07-07	7.19	0.54	165
1EX6	2000-05-01	1EX7	2000-05-01	4.37	0.78	281
1F3Y	2000-06-06	1JKN	2001-07-12	5.08	0.79	234
1FMF	2000-08-17	1ID8	2001-04-04	4.74	0.61	264
1GH1	2000-10-29	1CZ2	1999-09-01	3.04	0.68	173
1IJA	2001-04-25	2KID	2009-05-01	4.54	0.79	332
1JFJ	2001-06-20	1JFK	2001-06-21	14.73	0.47	227
1K2H	2001-09-27	1ZFS	2005-04-20	6.59	0.49	197
1GUD	2002-01-24	1RPJ	1999-02-04	4.45	0.72	409
1MO7	2002-09-08	1MO8	2002-09-08	4.93	0.79	297
1ORM	2003-03-14	1QJ8	1999-06-23	6.13	0.51	292
1WD7	2004-05-12	1WCW	2004-05-06	4.12	0.71	435
1W4U	2004-07-29	1UR6	2003-10-27	3.01	0.75	262
1VR6	2005-02-14	1RZM	2003-12-24	10.10	0.81	243
1Z15	2005-03-03	1Z17	2005-03-03	6.45	0.66	333
2CG7	2006-02-27	2CG6	2006-02-27	6.25	0.55	196
2NLN	2006-10-20	1RRO	1992-08-27	3.90	0.63	196
2UZ5	2007-04-25	2VCD	2007-09-20	3.37	0.78	201
2JU3	2007-08-14	2JU8	2007-08-15	3.17	0.73	160
2JWW	2007-10-25	1RTP	1993-05-14	2.79	0.73	191
2K43	2008-05-28	2K8R	2008-09-22	3.93	0.73	198
2KQ2	2009-10-24	2KW4	2010-03-31	14.14	0.28	325
2KXL	2010-05-10	2K0G	2008-02-02	6.09	0.66	254
2L50	2010-10-22	2L51	2010-10-22	4.56	0.73	196
2LKC	2011-10-10	2LKD	2011-10-10	7.47	0.72	244
4LP5	2013-07-15	4P2Y	2014-03-05	11.69	0.74	315

Table S3 | Impact of the different components on the structure modeling accuracy on the apo-holo benchmark.

	Ablation model	NMR fine-tuning	Heuristic iteration	Model integration	RMSD against apo state (Å)	RMSD against holo state (Å)
1)	baseline (trX2)				5.00	4.77
2)	baseline + NMR training set	√			4.94	4.17
3)	baseline + iteration		√		4.52	4.16
4)	baseline + NMR training set + iteration	√	√		4.53	3.79
5)	integration of (1) and (2)			√	4.37	3.84
6)	integration of (3) and (4) (trX2-D)	√	√	√	3.98	3.37

Table S4 | Comparison of AF2, AF-Cluster, AFsample2, trX2, and trX2-D on apo and holo proteins.

**Apo state**	**RMSD against apo state(Å)**					**Holo state**	**RMSD against holo state(Å)**				
**PDB ID**	**AF2**	**AF-Cluster**	**AFsample2**	**trX2**	**trX2-D**	**PDB ID**	**AF2**	**AF-Cluster**	**AFsample2**	**trX2**	**trX2-D**
2LAO	4.62	1.95	0.92	2.91	2.83	1LAH	0.41	0.51	0.47	3.00	1.59
1NTR	4.14	4.17	4.28	4.40	4.10	1KRX	3.25	3.25	3.31	3.22	2.92
1CFC	5.15	5.51	4.47	7.61	5.78	5DOW	5.73	2.62	4.34	8.41	6.72
1MUT	3.68	3.63	3.66	3.96	3.75	1PUN	3.75	3.68	3.82	4.26	3.90
1LIP	1.57	1.52	1.54	2.09	1.88	1JTB	2.91	2.77	2.84	2.82	2.63
4AKE	5.91	1.84	1.61	3.59	3.25	2ECK	0.79	1.92	1.98	5.10	1.79
1SYM	5.50	4.68	3.91	5.06	4.94	1XYD	1.96	5.13	2.06	3.48	3.43
1AEL	2.49	2.54	2.44	2.77	2.49	1URE	1.50	1.59	1.49	1.76	1.71
2CJO	1.29	1.48	1.38	1.72	1.34	1ROE	4.32	4.38	4.25	4.26	4.21
2RCS	5.06	3.95	1.84	7.57	3.43	1AJ7	1.96	8.23	2.19	9.50	4.60
1URP	3.72	0.79	0.84	2.75	2.47	2DRI	0.51	0.57	0.48	2.33	1.12
1SKT	4.40	2.21	2.78	3.93	2.87	1TNQ	3.00	3.29	2.98	4.00	3.80
1EX6	0.88	1.59	1.07	3.10	3.00	1EX7	1.04	1.70	1.55	2.07	1.63
1F3Y	3.01	3.62	2.74	4.32	3.80	1JKN	2.11	2.43	1.94	2.68	2.47
1FMF	1.84	2.28	1.84	2.49	2.19	1ID8	4.56	4.48	4.39	4.40	4.15
1GH1	2.28	1.98	2.10	2.18	1.99	1CZ2	2.65	2.55	2.57	2.53	2.48
1IJA	3.15	3.97	2.84	5.92	6.18	2KID	1.79	1.56	1.72	6.45	4.76
1JFJ	13.49	11.71	13.18	10.32	10.23	1JFK	12.86	12.92	12.91	11.52	6.38
1K2H	6.43	4.91	4.80	5.32	5.37	1ZFS	2.14	2.37	2.03	3.35	3.14
1GUD	3.63	1.07	0.98	3.58	3.35	1RPJ	0.92	1.18	1.11	2.90	2.77
1MO7	3.77	3.94	3.90	5.51	4.23	1MO8	3.40	3.51	3.24	4.67	4.04
1ORM	5.68	6.17	5.57	5.26	4.82	1QJ8	0.95	0.84	0.70	2.10	1.49
1WD7	1.99	4.72	4.49	14.24	4.33	1WCW	0.81	1.20	1.03	14.20	1.52
1W4U	2.24	2.24	2.21	2.26	2.04	1UR6	2.16	2.11	2.13	2.46	2.02
1VR6	1.38	6.27	1.65	6.17	4.11	1RZM	9.13	6.86	0.80	4.51	4.2
1Z15	6.07	5.25	4.16	4.74	4.50	1Z17	0.60	0.87	0.73	2.64	1.63
2CG7	0.93	3.33	1.08	6.45	2.16	2CG6	6.22	3.66	3.11	9.57	2.52
2NLN	3.91	3.95	3.86	3.61	3.43	1RRO	0.64	0.73	0.59	1.11	1.13
2UZ5	3.01	2.67	3.07	3.10	2.96	2VCD	1.38	1.57	1.40	1.86	1.50
2JU3	2.68	2.70	2.74	2.68	2.52	2JU8	1.76	1.79	1.77	1.71	1.71
2JWW	2.63	2.61	2.54	2.85	2.82	1RTP	0.38	0.50	0.44	1.42	1.45
2K43	2.76	3.04	2.81	2.80	2.67	2K8R	4.01	4.17	4.05	3.68	3.39
2KQ2	8.90	9.49	8.92	9.67	8.37	2KW4	10.70	7.14	9.50	9.13	8.54
2KXL	4.70	2.63	2.61	3.23	2.74	2K0G	3.32	3.20	3.23	3.88	3.23
2L50	3.09	4.58	3.02	10.13	5.10	2L51	3.27	5.30	3.52	9.88	4.70
2LKC	7.32	7.05	7.50	7.64	6.01	2LKD	3.98	3.75	3.92	5.05	4.56
4LP5	9.16	17.47	5.93	8.94	9.18	4P2Y	7.14	16.96	7.04	10.53	10.94
**Average**	**4.12**	**4.15**	**3.39**	**5.00**	**3.98**	**Average**	**3.19**	**3.55**	**2.85**	**4.77**	**3.37**

Table S5 | Information of 20 paired dual-conformation proteins from the Cfold benchmark set, the definitions of Fold1 and Fold2 are provided by Chakravarty D, *et al* [4].

**Fold1 state**		**Fold2 state**		**RMSD(Å) between Fold1 and Fold2**	**TM-score between Fold1 and Fold2**	**trX2-D sample number**
**PDB ID**	**Release Date**	**PDB ID**	**Release Date**			
1RLM	2003-11-26	2HF2	2006-06-22	3.58	0.79	299
2BBW	2005-10-17	2AR7	2005-08-19	5.16	0.75	301
2IEY	2006-09-19	2IEZ	2006-09-19	5.49	0.70	234
2NXE	2006-11-17	2ZBP	2007-10-26	11.75	0.78	368
2V9R	2007-08-25	2V9Q	2007-08-25	3.87	0.67	287
3BIS	2007-11-30	3BIK	2007-11-30	3.86	0.73	287
3PIL	2010-11-07	3PIM	2010-11-07	7.39	0.74	221
3T9L	2011-08-03	4A3P	2011-10-03	4.92	0.61	247
3UV5	2011-11-29	7L6X	2020-12-24	11.97	0.57	469
5JGK	2016-04-20	5JGL	2016-04-20	6.63	0.68	252
5O37	2017-05-23	5O2K	2017-05-21	6.81	0.58	271
5OVZ	2017-08-30	4P0I	2014-02-21	4.58	0.67	412
6DZX	2018-07-05	6CVA	2018-03-27	3.91	0.73	279
6HNI	2018-09-15	6HNK	2018-09-15	4.31	0.75	380
6P8R	2019-06-07	6P8O	2019-06-07	6.15	0.58	203
6SVF	2019-09-18	6GGP	2018-05-03	5.68	0.58	354
6UHI	2019-09-27	6UHS	2019-09-27	3.76	0.68	205
6ZJB	2020-06-28	6ZJD	2020-06-28	6.74	0.76	315
7RDT	2021-07-11	7RDS	2021-07-11	8.75	0.51	258
8DP6	2022-07-15	8DP7	2022-07-15	4.40	0.72	499

Table S6 | Comparison of Cfold (dropout), Cfold (noise), trX2, and trX2-D on 20 dual-conformation proteins.

**Fold1 state**	**RMSD against Fold1 state(Å)**				**Fold2 state**	**RMSD against Fold2 state(Å)**			
**PDB ID**	**Cfold (dropout)**	**Cfold (cluster)**	**trX2**	**trX2-D**	**PDB ID**	**Cfold (dropout)**	**Cfold (cluster)**	**trX2**	**trX2-D**
1RLM	1.02	1.04	1.51	1.53	2HF2	3.26	3.18	3.84	2.97
2BBW	3.87	3.50	2.93	2.56	2AR7	2.64	2.53	6.44	3.97
2IEY	15.99	15.91	15.98	15.61	2IEZ	17.50	17.46	17.48	17.01
2NXE	5.79	5.78	4.86	4.50	2ZBP	7.63	6.40	10.36	3.77
2V9R	2.41	2.17	2.46	2.22	2V9Q	4.92	4.28	5.20	3.67
3BIS	5.79	3.97	4.65	2.54	3BIK	3.35	2.25	3.61	3.07
3PIL	1.62	1.69	3.47	2.49	3PIM	6.95	6.93	7.76	7.12
3T9L	3.17	2.95	3.11	3.04	4A3P	1.77	1.56	4.09	3.55
3UV5	12.28	11.90	4.13	3.71	7L6X	1.45	1.43	13.69	3.40
5JGK	1.22	1.24	1.93	1.93	5JGL	5.82	5.98	7.51	6.65
5O37	1.25	1.28	1.86	1.74	5O2K	6.18	5.20	5.47	3.37
5OVZ	1.23	1.13	1.85	1.82	4P0I	3.06	3.05	3.83	3.47
6DZX	1.29	1.21	1.93	1.93	6CVA	2.95	2.95	3.71	3.59
6HNI	1.58	1.13	1.66	1.65	6HNK	4.05	3.76	4.04	3.74
6P8R	1.64	1.41	2.69	2.58	6P8O	4.42	4.44	5.54	4.61
6SVF	0.98	1.00	1.57	1.35	6GGP	5.20	4.93	5.72	5.12
6UHI	3.76	3.81	2.64	2.53	6UHS	3.20	3.31	3.64	2.31
6ZJB	5.07	4.83	1.66	1.53	6ZJD	1.96	1.91	6.07	3.42
7RDT	1.52	1.50	1.80	1.79	7RDS	8.43	8.47	8.14	8.09
8DP6	2.17	1.54	2.21	1.99	8DP7	1.52	1.60	2.70	2.20
**Average**	**3.68**	**3.45**	**3.24**	**2.95**	**Average**	**4.81**	**4.58**	**6.44**	**4.75**

Table S7 | Comparison between the input-driven strategies and trX2-D.

**Strategy**	**Key mechanism**	**Advantages**	**Disadvantages**
Input-driven (AF-Cluster, AFsample2)	Perturbing input MSAs (clustering or subsampling).	- Rapid end-to-end inference via GPU acceleration.<br>- Inherits high baseline accuracy from the AF2 architecture.	- Indirect output control limits the exploration of conformational space.<br>- Lacks mechanistic interpretability of the conformational transitions.<br>- Relies on the rich evolutionary information for effective clustering/sampling.
Output-driven (trX2-D)	Iteratively sampling predicted 2D geometries + energy minimization.	- Direct modulation of predicted geometries enables more effective capturing of alternative states.<br>- Provides physical interpretability via energy landscapes.<br>- Robust with sparse evolutionary information.	- Accuracy limited by baseline model.<br>- Lower throughput due to iterative CPU-based energy minimization.<br>- Relies on multi-state signals in predicted geometries.

Table S8 | Analysis of inference times. This comparison was performed on 20 targets with lengths ranging from 100 to 200 residues from the apo-holo dataset. The experiment is performed on a single CPU core.

PDB ID	Sequence length	Geometry prediction time (s)	Time per Rosetta iteration (s)	Total time(s)
1RRO	108	17.68	305.79	323.47
1RTP	109	17.88	319.41	337.29
1MUT	129	79.27	400.57	479.84
1URE	131	38.24	398.86	437.10
1QJ8	148	41.21	436.70	477.91
1EX7	186	146.35	620.82	767.16
1F3Y	165	118.77	534.31	653.08
1FMF	137	33.18	408.65	441.83
1IJA	148	36.20	452.53	488.73
1JFJ	134	88.00	328.91	416.91
1KRX	124	90.73	403.59	494.32
1W4U	147	103.76	487.06	590.82
2JU8	127	28.65	389.34	417.99
2VCD	137	92.03	456.83	548.86
2K0G	142	94.92	407.02	501.93
2K8R	133	87.61	435.21	522.82
2KQ2	147	55.38	486.69	542.07
2L51	102	17.89	219.95	237.85
2LKD	178	137.83	546.16	683.98
5DOW	146	101.87	349.58	451.45
**Average**	138.9	71.37	419.40	490.77

Table S9 | Information of 31 NMR proteins, along with the maximum pairwise RMSD(Å) and the minimum pairwise TM-score.

PDB ID	Release date	Maximum pairwise RMSD(Å)	Minimum pairwise TM-score	trX2-D sample number
1BZK	1998-11-01	8.94	0.24	141
1N0Z	2002-10-15	1.76	0.26	128
1W9R	2004-10-15	8.28	0.66	190
2A7Y	2005-07-06	2.59	0.73	150
2FFT	2005-12-20	36.54	0.25	162
2EOD	2007-03-29	1.79	0.72	156
2JU4	2007-08-14	13.02	0.08	167
2RNN	2008-01-30	9.69	0.83	109
2K4F	2008-06-07	12.98	0.22	148
2K9P	2008-10-21	7.62	0.48	194
2KEB	2009-01-28	2.96	0.74	183
2WCY	2009-03-17	3.24	0.79	227
2KLZ	2009-07-12	2.37	0.42	111
2KSD	2010-01-02	8.59	0.15	167
2KWQ	2010-04-15	2.91	0.79	157
2L7S	2010-12-21	14.17	0.41	132
2L8E	2011-01-11	6.58	0.67	131
2LFP	2011-07-07	3.43	0.78	233
2LTF	2012-05-22	10.66	0.89	134
2LXW	2012-09-03	2.01	0.74	145
2M2F	2012-12-20	2.42	0.42	222
2M3E	2013-01-17	2.54	0.79	125
2M6M	2013-04-06	2.66	0.77	207
2M9U	2013-06-19	3.40	0.73	88
2MXV	2015-01-16	1.33	0.64	156
2NDJ	2016-06-09	19.13	0.27	203
5KZO	2016-07-25	5.77	0.57	149
5M4T	2016-10-19	10.62	0.61	181
5NAM	2017-02-28	0.79	0.75	132
6NU4	2019-01-30	20.93	0.31	198
6UCH	2019-09-16	0.53	0.66	112

Table S10 | Summary of data redundancy removal strategies for 3 benchmark sets.

Benchmark dataset	Size	Redundancy removal strategy
Apo-holo pairs	37	Sequences sharing >50% sequence identity with these test targets were excluded from the training and fine-tuning set
Cfold dual-conformation proteins	20	Filtered with a 40% sequence identity threshold against the training and fine-tuning set.
NMR dynamic proteins	31	Proteins sharing >30% sequence identity relative to the training and fine-tuning dataset were excluded.

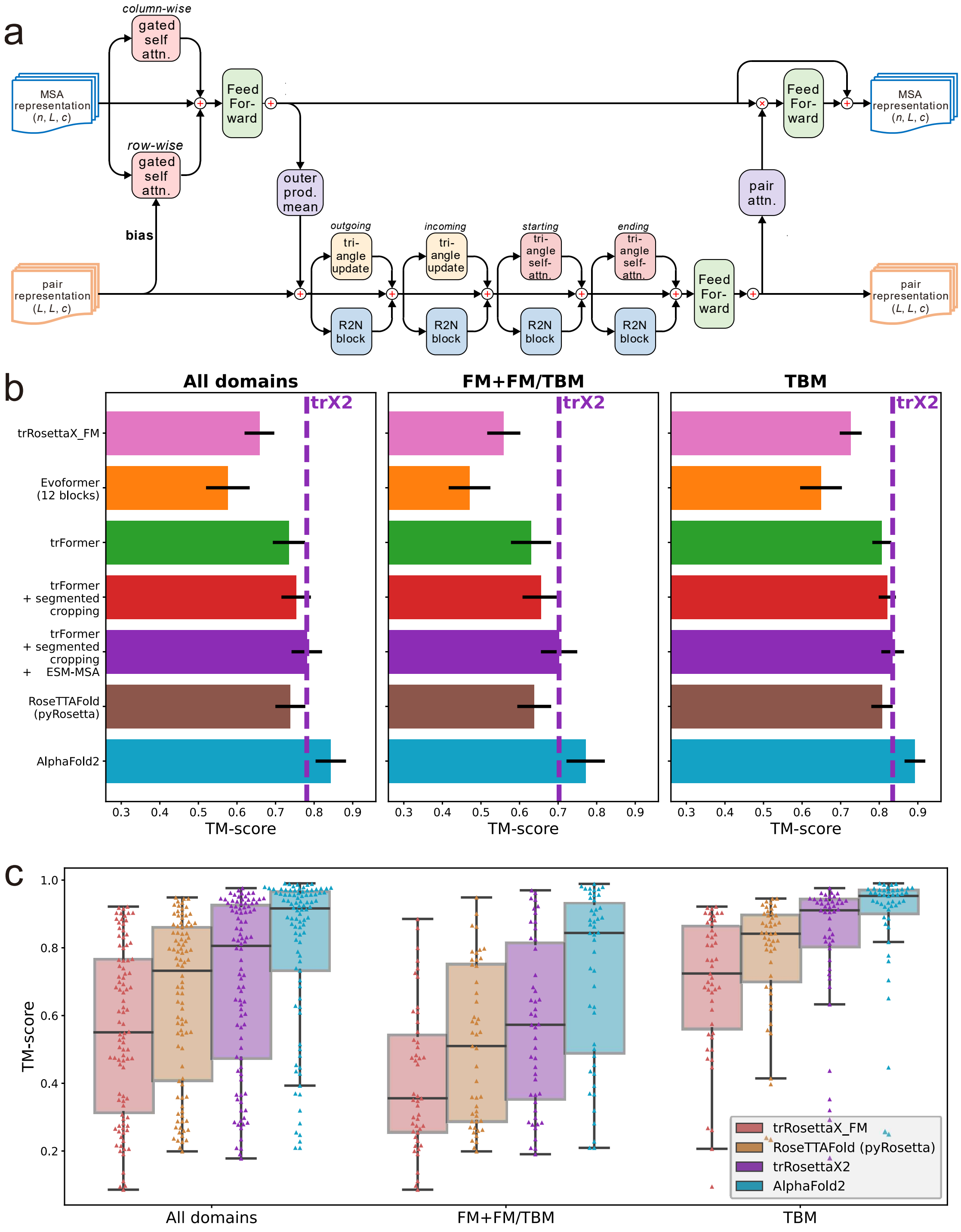

Fig. S1 | Architecture and performance of trRosettaX2. (a) architecture of a trFormer block. **(b)** average TM-scores on CASP14 domains for trRosettaX_FM, trRosettaX2 and its variants, RoseTTAFold, and AlphaFold2. Error bars represent one-fifth of the standard deviation. **(c)** TM-score distributions on CASP15 domains.

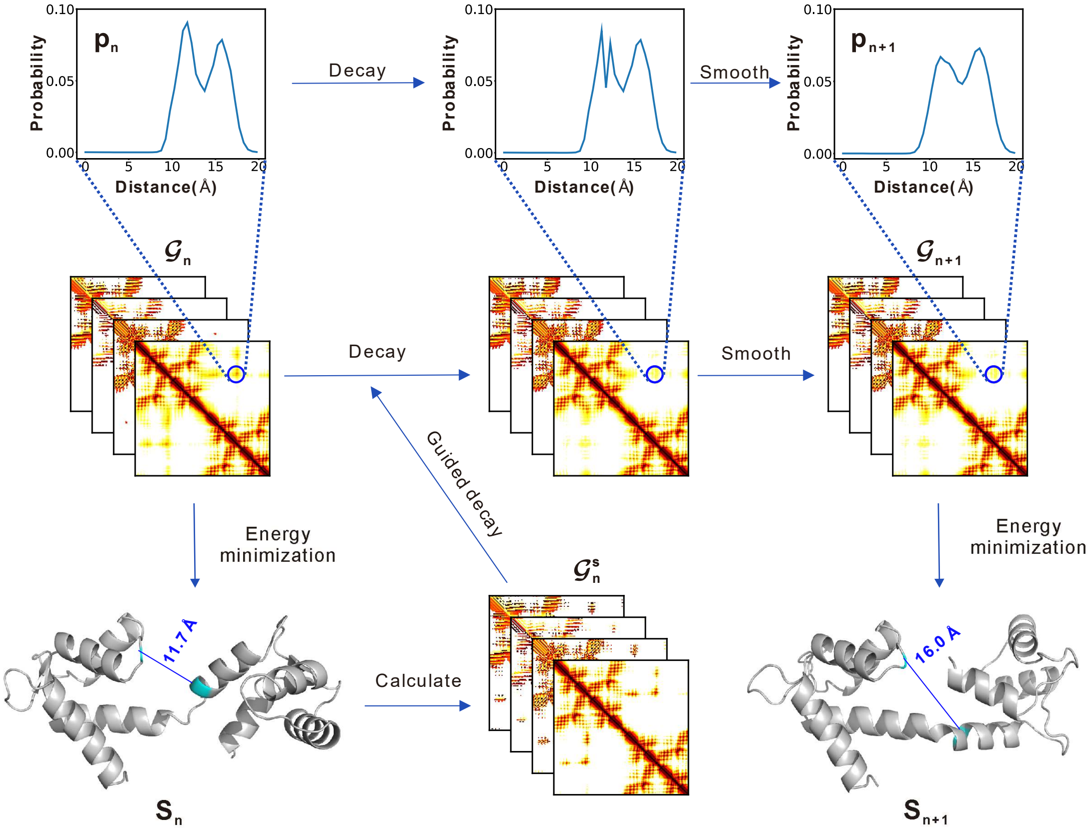

Fig. S2 | Illustration of one iteration of the heuristic iterative sampling process in trX2-D.

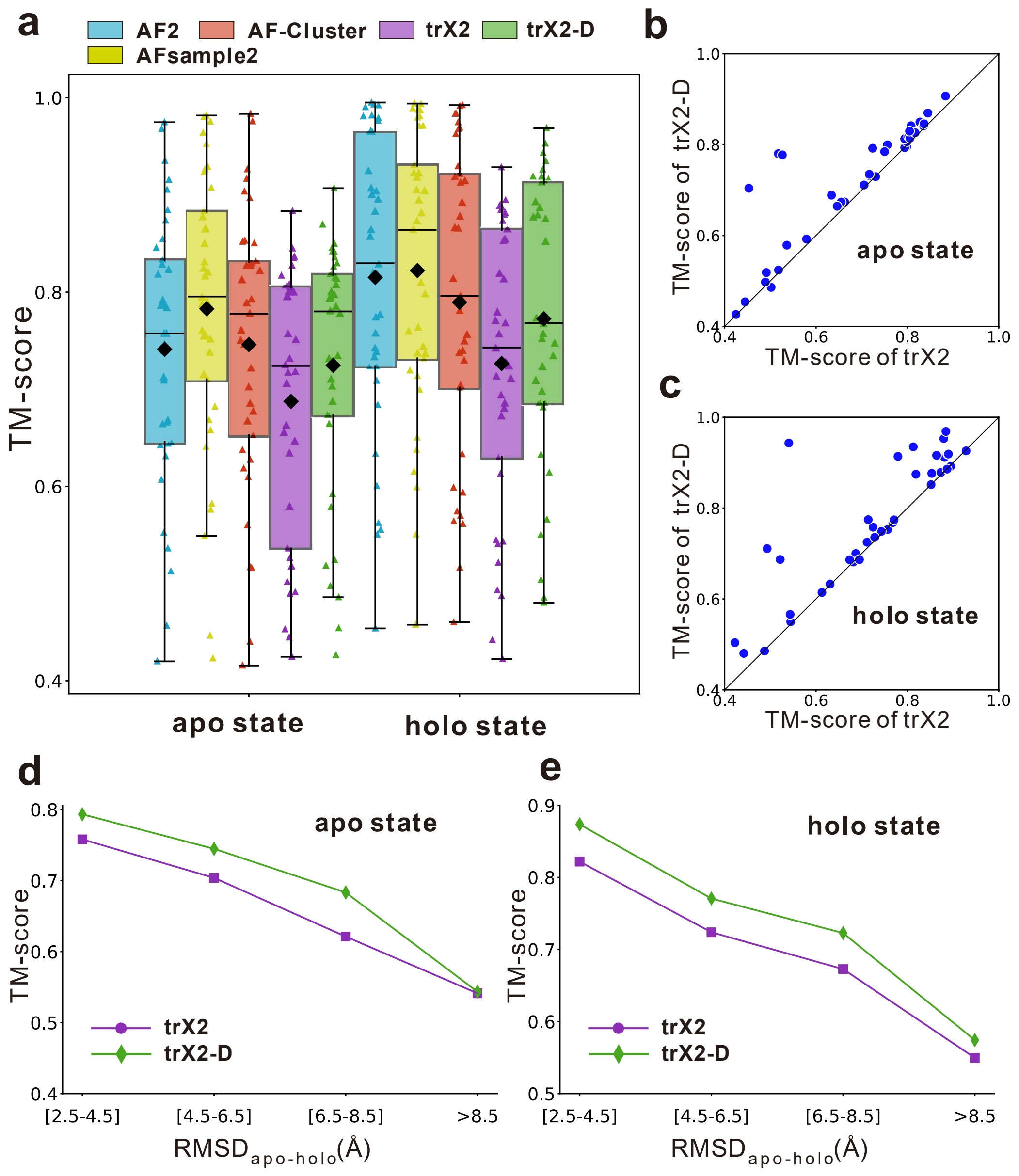

Fig. S3 | TM-score comparison of trX2-D on proteins with apo and holo states. (a) box plots illustrating the TM-score distribution between predicted and native structures for AF2, AF-Cluster, AFsample2, trX2, and trX2-D, stratified by apo and holo states. Mean TM-scores are indicated by black diamond. **(b, c)** head-to-head TM-score comparison between trX2-D and trX2 for the apo state (b) and holo state (c). **(d, e)** average TM-scores of trX2 and trX2-D predictions for the apo (d) and holo (e) states, grouped by the $\text{RMSD}_{\text{apo-holo}}$.

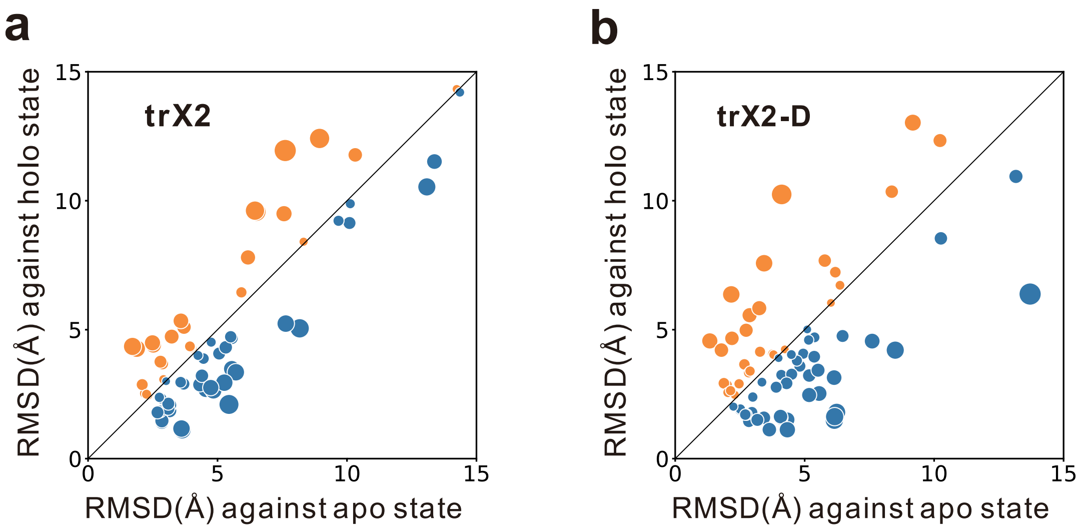

Fig. S4 | Comparison of RMSDs relative to native apo/holo states for the predicted conformations by trX2 (a) and trX2-D (b). Point size is proportional to the perpendicular distance to the diagonal.

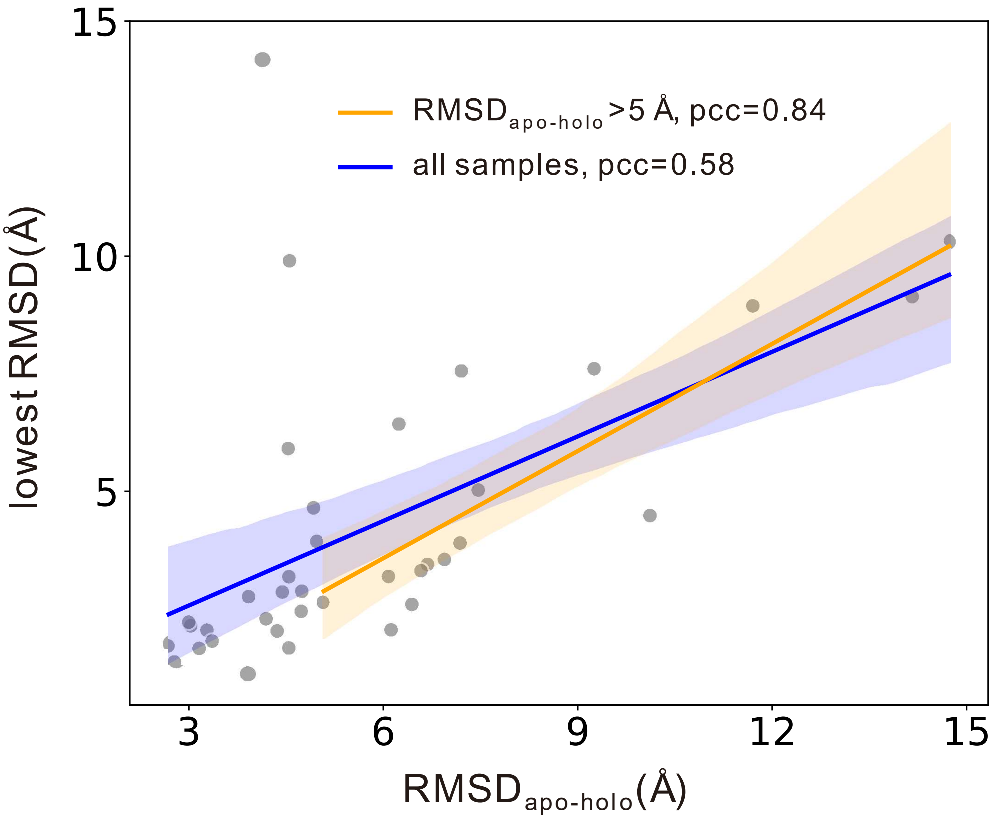

Fig. S5 | Correlation between native state divergence (RMSD$_{apo-holo}$) and trX2 prediction accuracy (the minimum RMSD to any state).

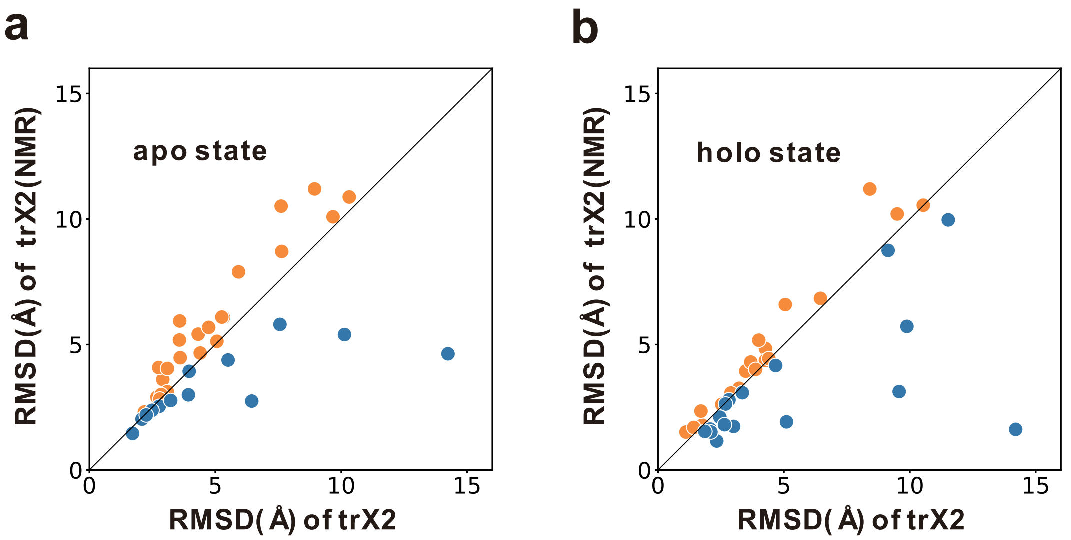

Fig. S6 | Head-to-head RMSD comparison between trX2 (NMR) and trX2 for the apo state (a) and the holo state (b).

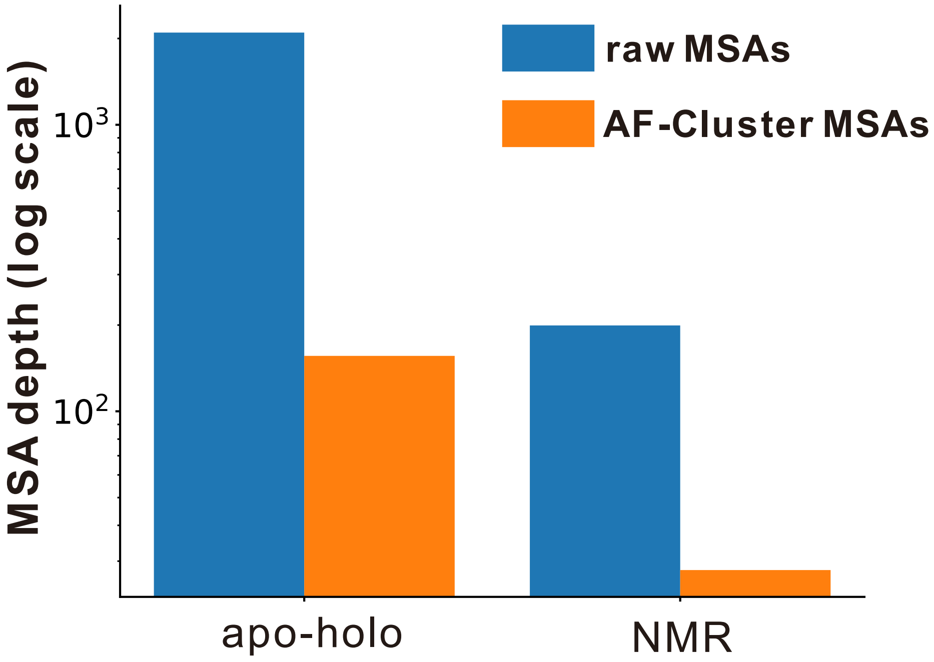

Fig.S7 | Comparison of MSA depths. Bar chart quantifying the mean MSA depth for raw sequences (blue) versus clustered sequences (orange) in the apo-holo and NMR datasets. The value for "AF-Cluster MSAs" is calculated as the average depth of the deepest MSA generated by AF-Cluster for each target across the respective datasets.

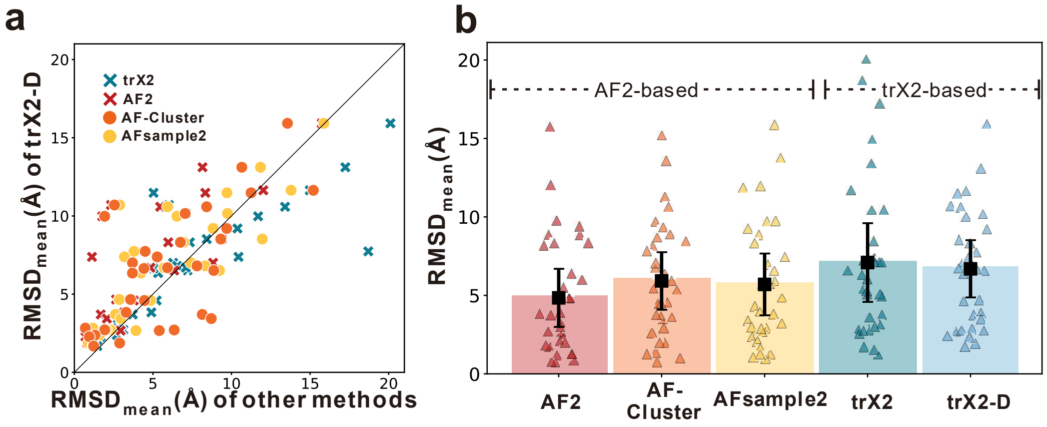

Fig. S8 | Comparison of the ensemble structural quality. (**a**) head-to-head comparisons of $RMSD_{mean}$ for trX2-D versus AF2, AF-Cluster, AFsample2, and trX2. (**b**) bar chart illustrating the average $RMSD_{mean}$ distributions for different methods. Unlike the degradation trend observed in AF2-based sampling, trX2-D maintains performance comparable to trX2.

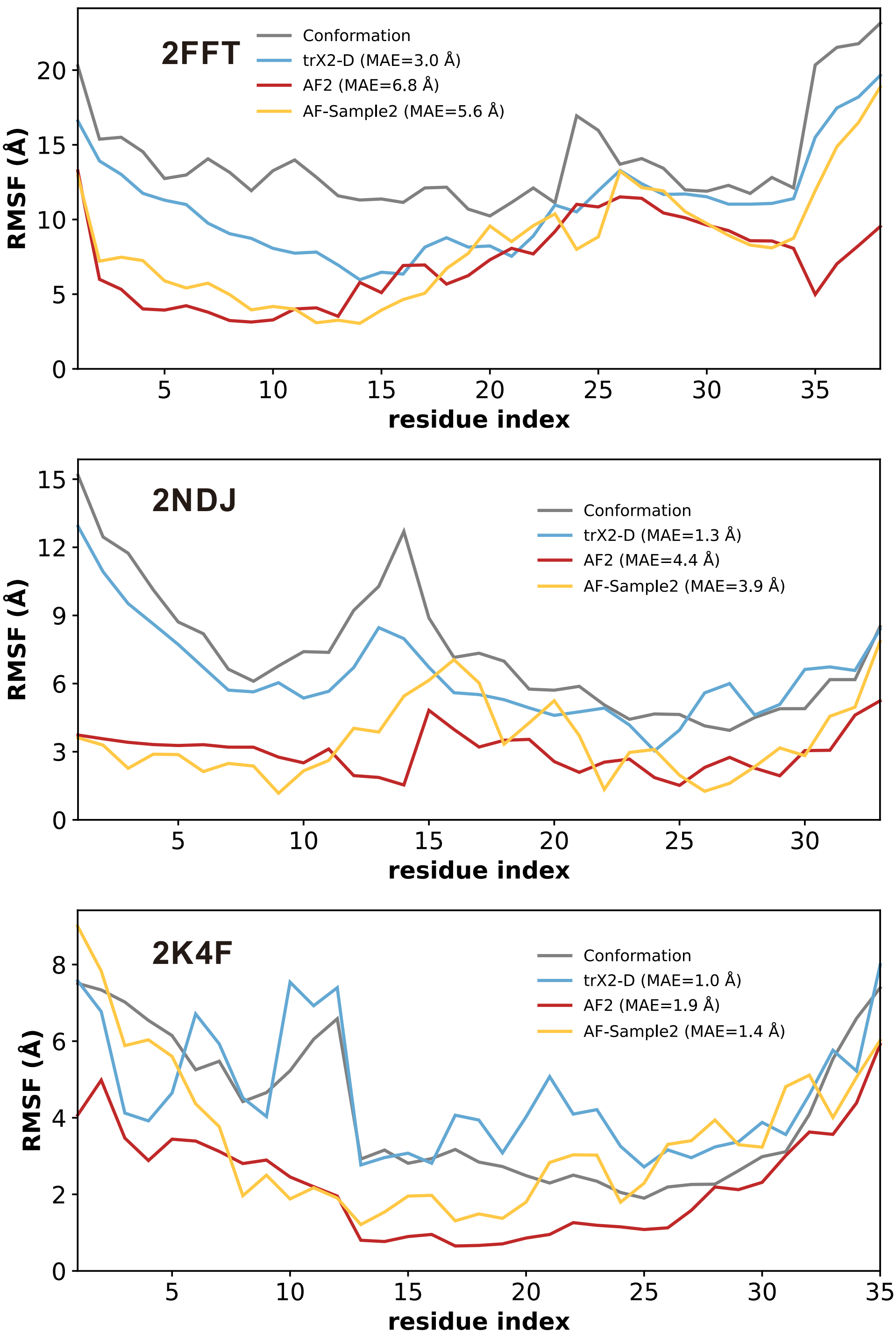

Fig. S9 | Per-residue RMSF profiles for three challenging cases shown in Fig. 6. Root-mean-square fluctuations (RMSF) are plotted against residue index for the native ensemble (grey), AlphaFold2 (red), AFsample2 (orange), and trX2-Dynamics (blue). Compared to AF2 and AFsample2, trX2-Dynamics captures broader structural diversity with RMSF more closely matching the native flexibility.

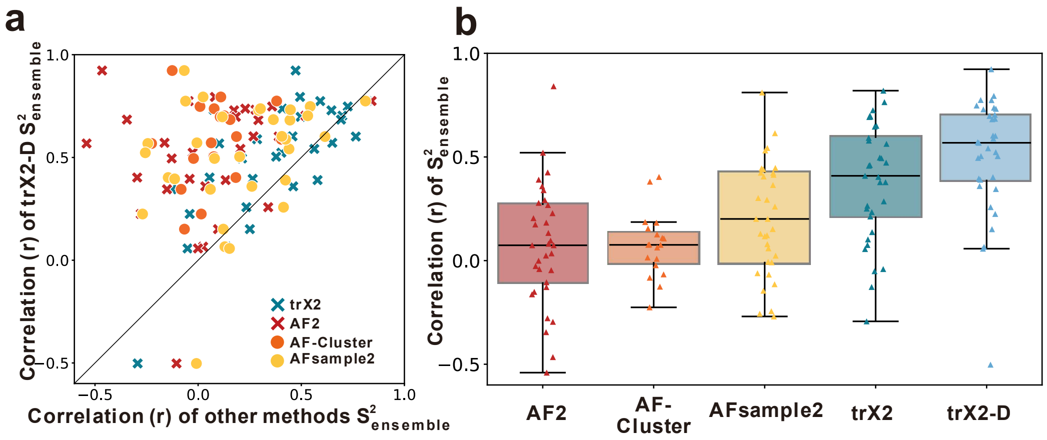

Fig. S10 | Performance of trX2-D and other methods on NMR backbone dynamics. (**a**) head-to-head comparisons of Pearson correlation (r) of $S^2_{ensemble}$ for trX2-D versus AF2, AF-Cluster, AFsample2, and trX2; points above the diagonal denote trX2-D superiority. (**b**) boxplots illustrating the distributions of correlation coefficients (r) for different methods on the NMR dataset.

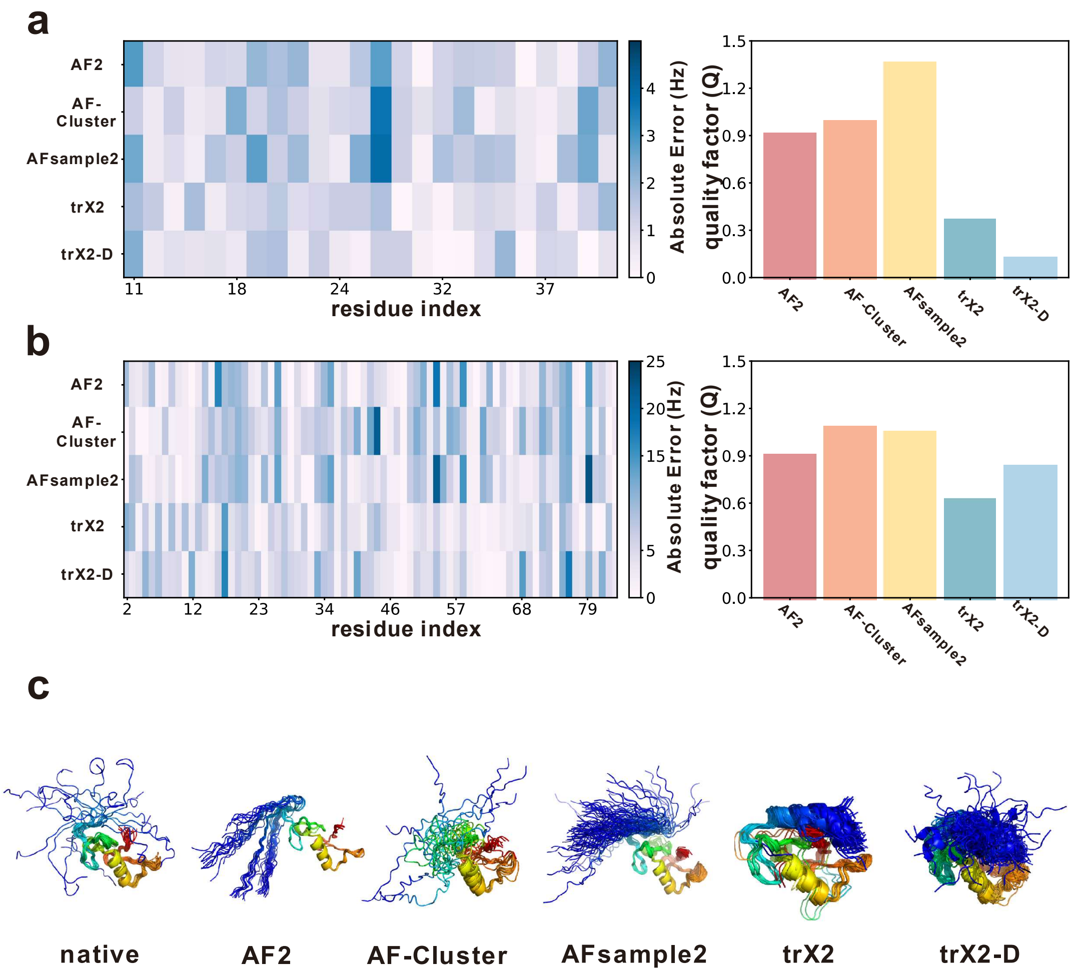

Fig. S11 | RDC analysis for the computational ensembles. (a, b) per-residue absolute error heatmaps (left) and global Q-factors (right) for targets 2M3E (a) and 2M6M (b). Lower Q-factors denote better agreement with experimental data. **(c)** visualization of experimental and predicted ensembles for 2M6M. The observed discrepancies highlight a common limitation across all methods in accurately capturing the precise spatial orientation of flexible fluctuations relative to the protein core.

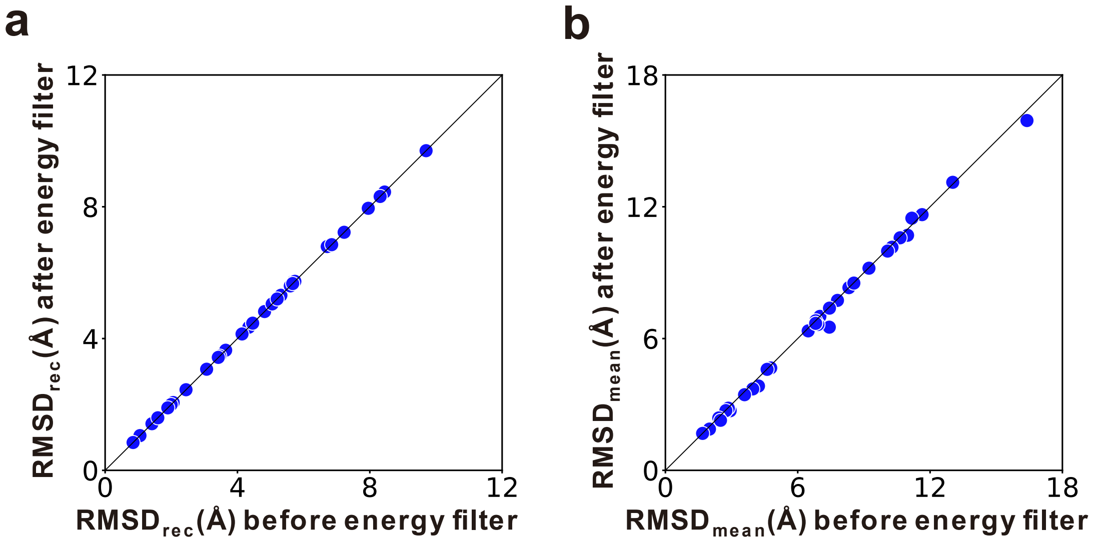

Fig. S12 | Impact of energy filtering on trX2-D performance on NMR benchmark set. Comparison of trX2-D results before and after energy-based filtering for $RMSD_{rec}$ (a) and $RMSD_{mean}$ (b). Notably, the filtering step improves the ensemble quality for 90.3% (28/31) of the targets.

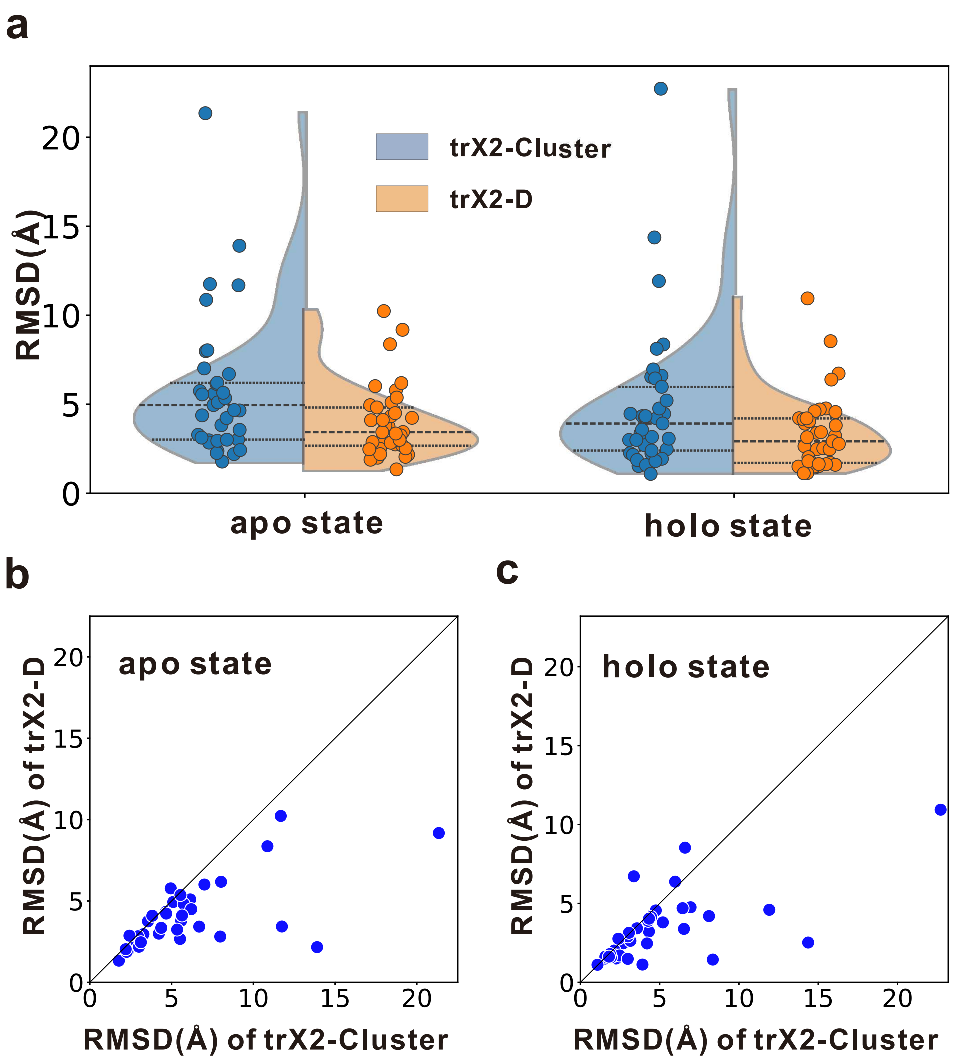

Fig. S13 | Comparison between trX2-D and trX2-Cluster. (a) violin plots comparing the distribution of RMSD values between predicted and native structures for trX2-Cluster and trX2-D in both apo and holo states. Individual data points are overlaid on the violins. **(b, c)** head-to-head comparisons of RMSD between trX2-D and trX2-Cluster for the apo state (b) and the holo state (c).

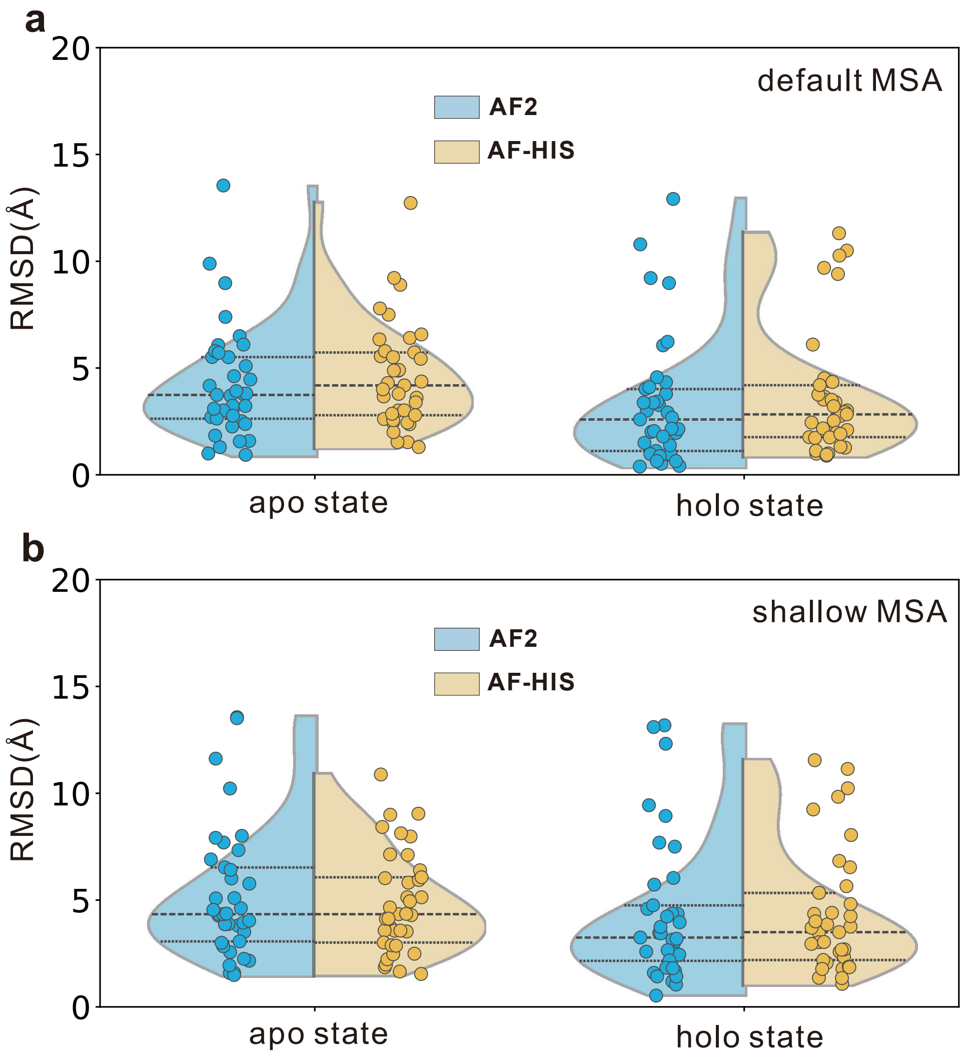

Fig. S14 | Applying iterative sampling to AF2 with different MSA depths. (a) violin plots showing the distribution of RMSD values for AF2 applying the heuristic iterative sampling strategy (AF-HIS) with default MSA depth, compared to standard AF2 predictions for both apo and holo states. **(b)** similar violin plots showing the impact of iterative sampling on AF2 predictions generated using shallow MSAs, compared to standard shallow MSA AF2 predictions. Individual data points are overlaid on all plots.

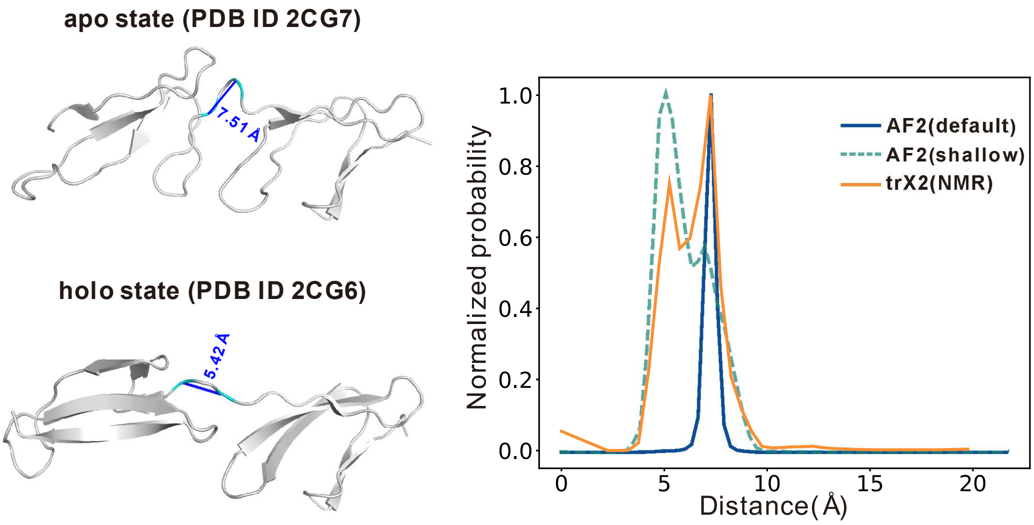

Fig. S15 | Comparison of distance distributions for a representative residue pair in apo and holo states. The left panels illustrate the apo (PDB ID 2CG7) and holo (PDB ID 2CG6) structures (cartoon representation), highlighting the residue pair and the corresponding distances in blue. The right panels present the normalized probability distributions of the distance between this pair predicted by trX2(NMR), AF2, and AF2 with subsampled MSA (AF2(shallow)).

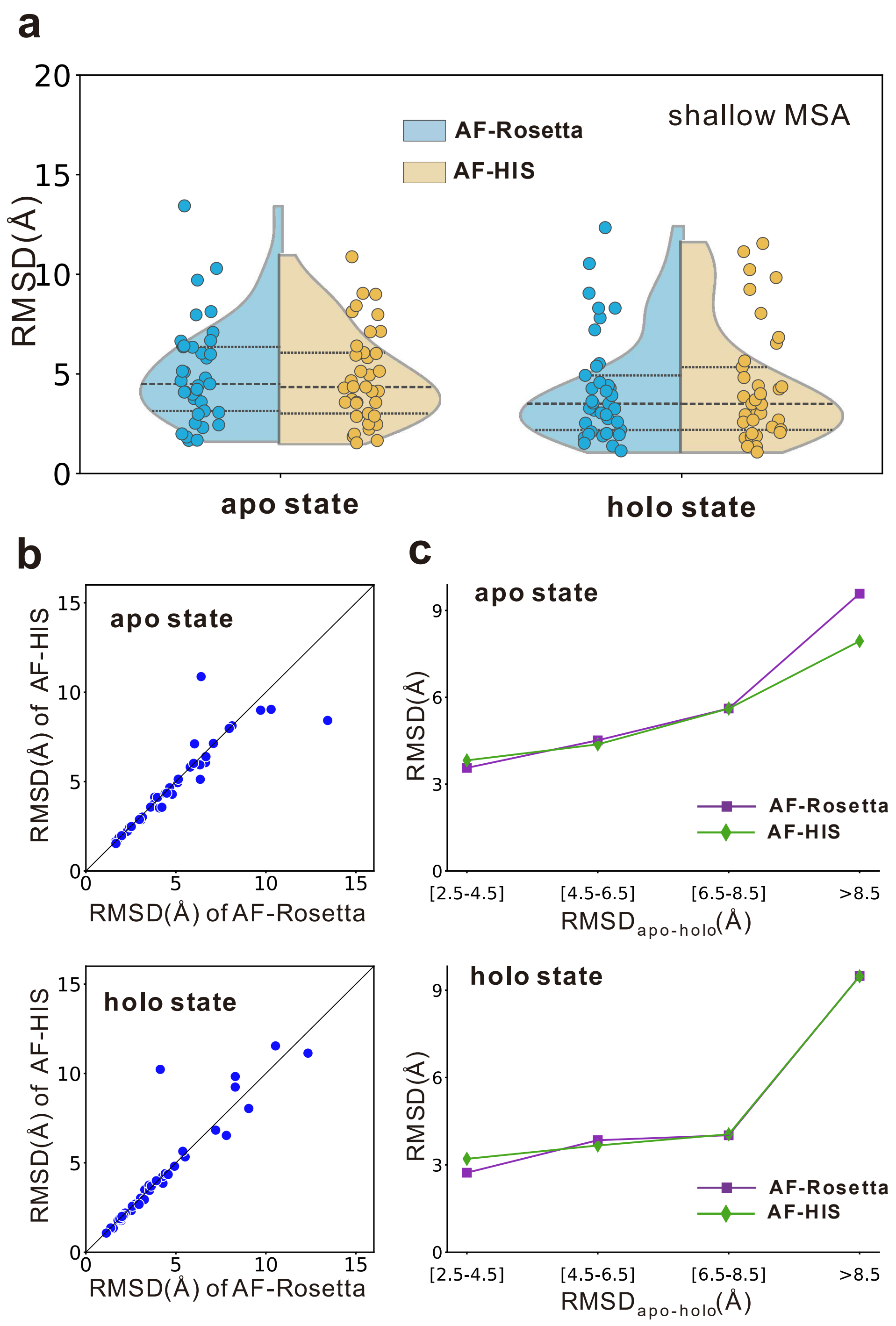

Fig. S16 | Comparison between AF-Rosetta and AF-HIS. (a) violin plots of RMSD distributions relative to native structures for both apo and holo states, with individual data points overlaid. **(b, d)** head-to-head RMSD comparisons for the apo state (b) and the holo state (d). **(c, e)** average RMSD values for the apo state (c) and the holo state (e), grouped by RMSD between native conformations ($RMSD_{apo\text{-}holo}$).

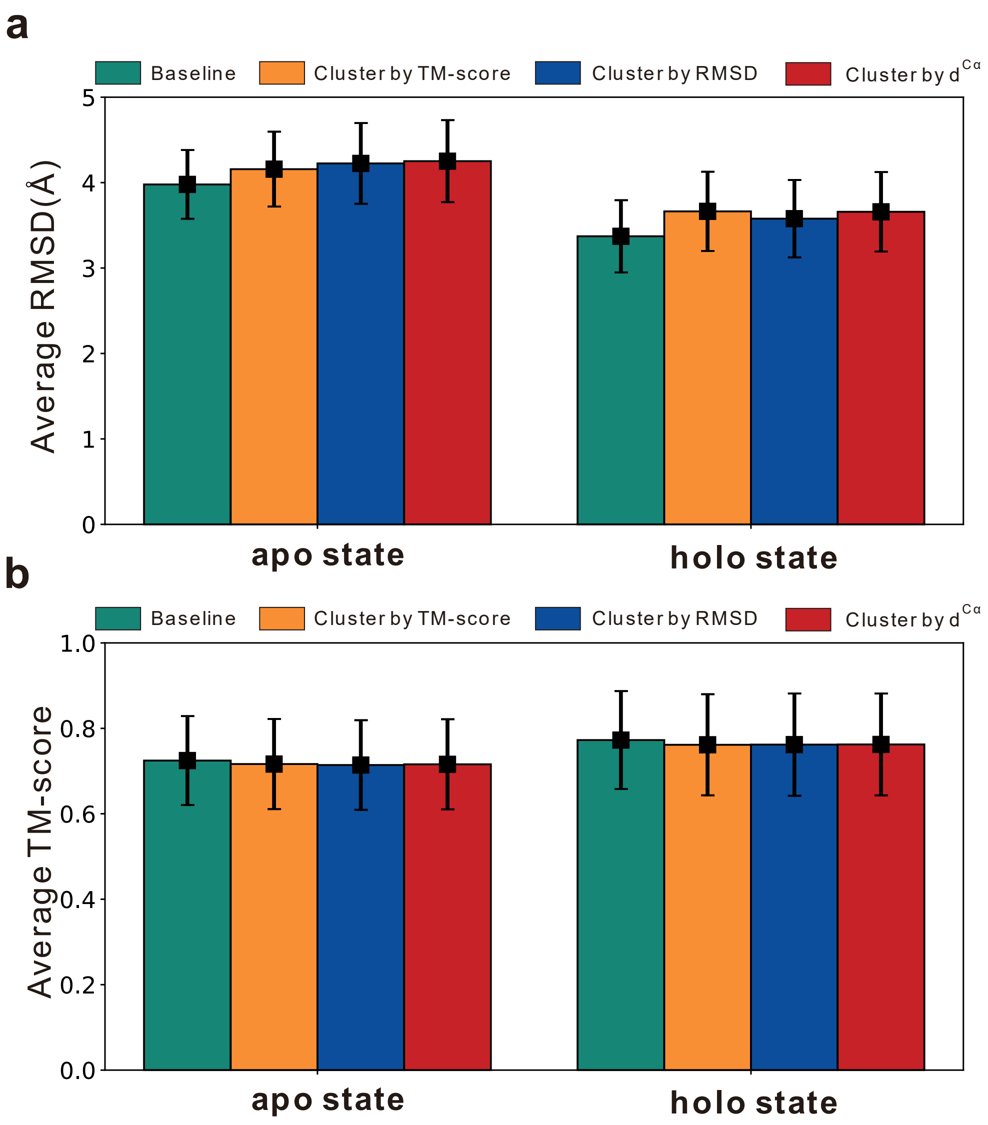

Fig. S17 | Evaluation of clustering strategies for representative structure selection. **(a, b)** the average RMSD (a) and TM-score (b) of representative structures selected using k-means clustering based on different structural similarity metrics (TM-score, RMSD, and inter-Cα distances $d^{C\alpha}$) for both apo and holo states. A baseline representing the average performance without clustering is also shown. Each error bar represents one-fifth of the standard deviation.

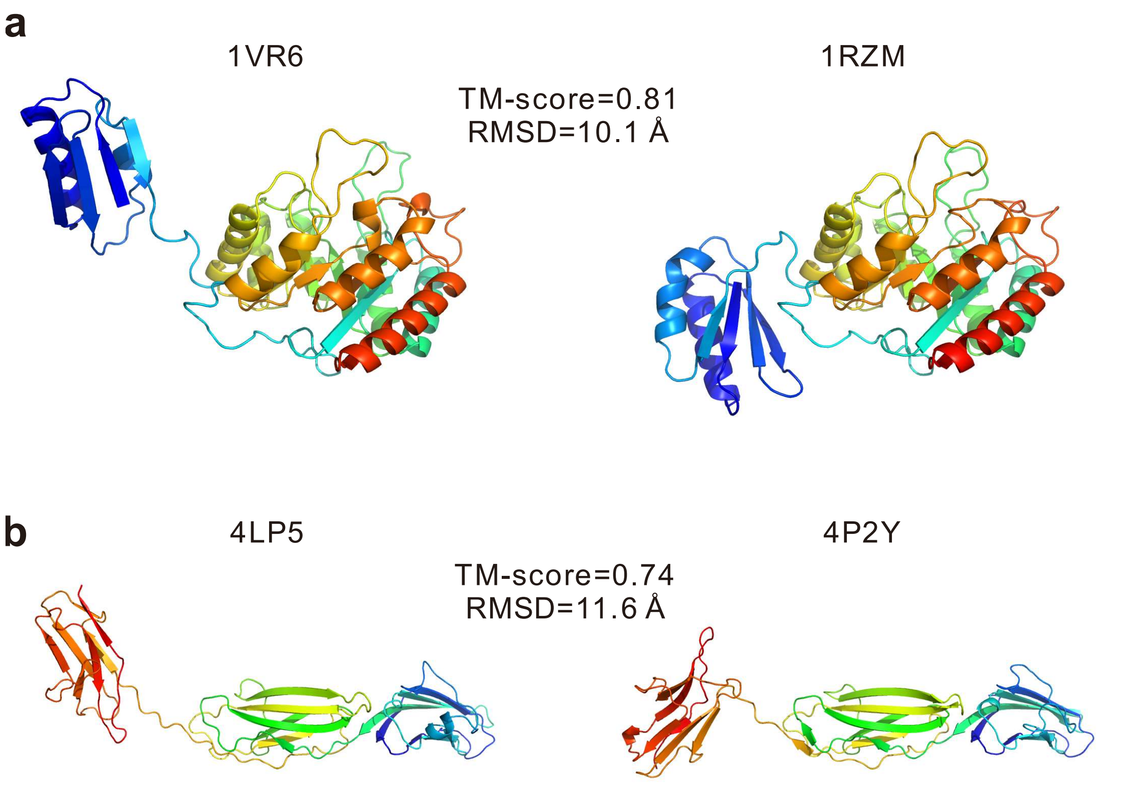

Fig. S18 | Examples of apo-holo protein pairs exhibiting significant conformational differences despite having high inter-conformation TM-scores.

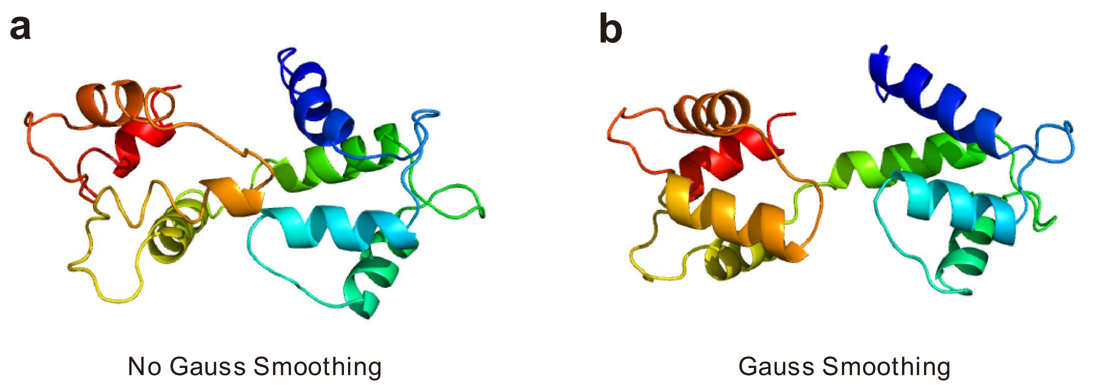

Fig. S19 | Visualizing the impact of Gaussian smoothing on protein structure representation. Representative protein structures are shown (a) without and (b), with Gaussian smoothing applied. Gaussian smoothing enhances structural regularity and completeness in the heuristic iterative process.

Supplementary References

1. Rao RM*, et al.* MSA Transformer. In: *Proceedings of the 38th International Conference on Machine Learning* (eds Marina M, Tong Z). PMLR (2021).

2. Gao SH, Cheng MM, Zhao K, Zhang XY, Yang MH, Torr P. Res2Net: A New Multi-Scale Backbone Architecture. *IEEE Transactions on Pattern Analysis and Machine Intelligence* **43**, 652-662 (2021).

3. Su H*, et al.* Improved Protein Structure Prediction Using a New Multi-Scale Network and Homologous Templates. *Advanced Science* **8**, 2102592 (2021).

4. Chakravarty D*, et al.* AlphaFold predictions of fold-switched conformations are driven by structure memorization. *Nat Commun* **15**, 7296 (2024).
